# Doxorubicin-induced neurotoxicity differently affects the hippocampal formation subregions in adult mice

**DOI:** 10.1016/j.heliyon.2024.e31608

**Published:** 2024-05-24

**Authors:** Ana Dias-Carvalho, Mariana Ferreira, Ana Reis-Mendes, Rita Ferreira, Maria de Lourdes Bastos, Eduarda Fernandes, Susana Isabel Sá, João Paulo Capela, Félix Carvalho, Vera Marisa Costa

**Affiliations:** a Associate Laboratory i4HB - Institute for Health and Bioeconomy, Faculty of Pharmacy, University of Porto, 4050-313, Porto, Portugal; b UCIBIO–Applied Molecular Biosciences Unit, Laboratory of Toxicology, Department of Biological Sciences, Faculty of Pharmacy, University of Porto, 4050‐313, Porto, Portugal; c LAQV/REQUIMTE, Chemistry Department, University of Aveiro, Aveiro, Portugal; d LAQV/REQUIMTE, Laboratory of Applied Chemistry, Department of Chemical Sciences, Faculty of Pharmacy, University of Porto, Porto, Portugal; e Unit of Anatomy, Department of Biomedicine, Faculty of Medicine, University of Porto, Porto, Portugal; f Center for Health Technology and Services Research (CINTESIS), Faculty of Medicine, University of Porto, Porto, Portugal; g FP-I3ID, Faculdade de Ciências da Saúde, Universidade Fernando Pessoa, Porto, Portugal

**Keywords:** Doxorubicin, Chemobrain, Hippocampus, Chemotherapy, Mice, Apoptosis

## Abstract

Doxorubicin (DOX) is an anthracycline used to treat a wide range of tumours. Despite its effectiveness, it is associated with a long range of adverse effects, of which cognitive deficits stand out. The present study aimed to assess the neurologic adverse outcome pathways of two clinically relevant cumulative doses of DOX. Adult male CD-1 mice received biweekly intraperitoneal administrations for 3 weeks until reaching cumulative doses of 9 mg/kg (DOX9) or 18 mg/kg (DOX18). Animals were euthanized one week after the last administration, and biomarkers of oxidative stress and brain metabolism were evaluated in the whole brain. Coronal sections of fixed brains were used for specific determinations of the prefrontal cortex (PFC) and hippocampal formation (HF). In the whole brain, DOX18 tended to disrupt the antioxidant defences, affecting glutathione levels and manganese superoxide dismutase expression. Considering the regional analysis, DOX18 increased the volume of all brain areas evaluated, while GFAP-immunoreactive astrocytes decreased in the dentate gyrus (DG) and increased in the CA3 region of HF, both in a dose-dependent manner. Concerning the apoptosis pathway, whereas Bax increased in the DOX9 group, it decreased in the DOX18 group. Only in the latter group did Bcl-2 levels also decrease. While p53 only increased in the CA3 region of the DOX9 group, AIF increased in the PFC and DG of DOX18. Finally, phosphorylation of Tau decreased with the highest DOX dose in DG and CA3, while TNF-α levels increased in CA1 of DOX18. Our results indicate new pathways not yet described that could be responsible for the cognitive impairments observed in treated patients.

## Abbreviations

AIF: apoptosis-inducing factor
ATP: adenosine triphosphate
BBB: blood-brain barrier
Bcl-2: B-cell lymphoma 2
DG: dentate gyrus
DOX: doxorubicin
DOX18: total cumulative dose of 18 mg/kg DOX
DOX9: total cumulative dose of 9 mg/kg DOX
eNOS: endothelial nitric oxide synthase
GFAP: glial fibrillary acidic protein
GSH: reduced glutathione
GSK-3β: glycogen synthase kinase 3 beta
GSSG: oxidized glutathione
HF: hippocampal formation
HSP27: heat shock protein 27
i.p.: intraperitoneal
ir: immunoreactive
MnSOD: manganese superoxide dismutase
PFC: prefrontal cortex
pTau: phosphorylated tau
tGSH: total glutathione
TNF-α: tumour necrosis factor alpha

## 1 Introduction

Cancer is a leading cause of premature death in most countries, and its incidence is expected to increase, reaching 28.4 million cases in 2040. That represents a 47 % increase from 2020 [1]. Nevertheless, early diagnosis and combination treatment also significantly increased the survival rate. Chemotherapy has been pivotal for the increase in cancer survival rates throughout the decades [2]. In fact, the cancer survivorship rate will continue to rise, being projected to increase by 54 % until 2040 in the United States of America [3]. Despite the high rates of success, chemotherapy presents several short- and long-term adverse effects that negatively affect the health and quality of life of cancer survivors. At present, more attention has been given to the neurotoxic effects developed during or after chemotherapy. Chemotherapy-induced cognitive impairment or ‘chemobrain’ has been established as a secondary effect of chemotherapy treatment and it may persist for the following years, with a prevalence between 28 % [4] and 75 % [5]. The cognitive domains reported more often to be impaired in those clinical studies were verbal memory, psychomotor function [6], visual memory, visuospatial and verbal learning [7], memory function and attention [8], suggesting extensive impairment mainly to the prefrontal cortex (PFC) and hippocampal formation (HF) [9].

Doxorubicin (DOX) is one of the most frequently used chemotherapeutic agents in cancer therapy. It is an anthracycline that presents several mechanisms of action, mainly inhibition of topoisomerase II, and binding and intercalation with the DNA, but it is also responsible for the overproduction of reactive species and interference with iron metabolism (thus favouring oxidative stress) [10,11]. It is used in a wide range of solid tumours, including breast, prostate, uterus, ovary, oesophagus, liver, bile ducts and stomach tumours, paediatric solid tumours, soft tissue sarcomas, multiple myeloma, Wilms’ tumour, and haematological malignancies [12]. DOX is either administered alone or as part of multiple anticancer regimens. Usually, the intravenous administration schedule is 60–75 mg/m^2^ every 3 weeks, depending on the tumour, but should not surpass the total lifetime cumulative dose of 400–550 mg/m^2^ [13]. Despite contributing to the high rates of success in cancer treatment, DOX is non-selective for cancer cells, so it causes toxicity in non-target organs [10].

The brain is mostly protected by the physical and functional blood-brain barrier (BBB), which is believed to impair the entrance of most chemotherapeutic agents. DOX is a substrate of the efflux pump P-glycoprotein, which highly restricts its entrance in the brain [14]. Thus, neither DOX nor its main metabolite, doxorubicinol, are detected in significant amounts in the brain after systemic administration [15], with the exception of when delivered in nanosystems [16]. Even so, DOX neurotoxicity caused by direct entrance seems highly unlikely, and it has been widely accepted that DOX neurotoxicity and resulting cognitive impairment is probably due to an indirect mechanism, i.e. peripheral toxicity or reactivity of DOX that culminates in neuronal damage. As mentioned above, DOX is a well-established inducer of oxidative stress due to its ability to undergo redox cycling generating reactive oxygen/nitrogen species or impacting iron homeostasis, which causes oxidative damage in several non-target organs [10]. DOX can evoke oxidative damage to plasma proteins, leading to an increase in the levels of the proinflammatory cytokine tumour necrosis factor alpha (TNF-α) [17,18]. This cytokine can readily cross tissue barriers, including the BBB and stimulate a cascade of local inflammation and oxidative stress [15,19]. To evaluate DOX-induced neurotoxicity and its link to TNF-α levels, Ren and colleagues [19] used a TNF-α knockout and wild type mice administered a with high dose of DOX (25 mg/kg). They observed increased plasma protein and lipid oxidation in both the TNF-α knockout and wild-type mice administered with DOX. On the other hand, when analysing changes in protein or lipid oxidation in the brain, the DOX-treated TNF-α knockout mice had similar values as the TNF-α knockout saline controls, while the DOX-treated wild-type had increased lipids and proteins oxidation compared to saline-treated wild-type controls. These data indicate that TNF-α is a mediator for the oxidation of brain macromolecules [19]. In a redox proteomics analysis, Aluise and colleagues determined that the increase in plasma TNF-α is due to the oxidation of plasma Apolipoprotein A1 , an endogenous inhibitor of TNF-α production by the macrophages [18]. Noteworthy, an increase in circulating pro-inflammatory cytokines was observed in chemotherapy-treated patients [20]. These cytokines can reach the brain and activate the microglia to produce other pro-inflammatory cytokines and other inflammatory mediators harmful to the brain [21]. The onsite inflammation and oxidative stress cause a downstream of events that can culminate in mitochondrial dysfunction and glial cell activation, which can trigger programmed cell death and proteasomal malfunction [22]. Despite all the available data, several questions remain unanswered, mainly referring to the intracellular mechanisms involved and whether those changes are seen in the multiple dose administration schedule at clinically relevant doses. Therefore, this work aimed to assess the possible differential neurotoxic effects of two clinically relevant cumulative doses of DOX in male CD-1 mice and clarify the possible intracellular mechanisms involved. In addition, an in-depth analysis was done in the PFC and hippocampal brain areas, which are more frequently described as malfunctioning.

## 2 Materials and methods

### 2.1 Materials

Sodium hydroxide was obtained from VWR chemicals (Leuven, Belgium), sodium carbonate from Panreac (Barcelona, Spain) and monosodium phosphate from J.T. Baker (Deventer, Holland). Perchloric acid (HClO_4_), potassium hydrogen carbonate and 4′,6-diamidino-2-phenylindole (DAPI) were obtained from Fisher scientific (Loughborough, United Kingdom). Copper (II) sulphate, ethylenediaminetetraacetic acid, Triton X-100, paraformaldehyde, immersion oil and FluorSave™ were obtained from Merck (Darmstadt, Germany). Isoflurane was obtained from Abbott Animal Health (North Chicago, IL, USA). Mouse monoclonal anti-ATP synthase β (ab14730), mouse monoclonal anti-endothelial nitric oxide synthase (eNOS) (ab76198), rabbit polyclonal anti-manganese superoxide dismutase (MnSOD) (ab13533), and rabbit polyclonal anti-glyceraldehyde 3-phosphate dehydrogenase (GAPDH) (ab9485) were obtained from Abcam (Cambridge, UK). Rabbit polyclonal anti-heat shock protein 27 (HSP27) (sc-9012), mouse monoclonal anti-B-cell lymphoma 2 (Bcl-2) antibody (sc-7382), rabbit polyclonal anti-Bax (sc-493), mouse monoclonal anti-apoptosis-inducing factor (AIF) antibody (sc-13116), and rabbit polyclonal anti-p53 (sc-6243) were obtained from Santa Cruz Biotechnology (Heidelberg, Germany). Rabbit polyclonal anti-glycogen synthase kinase 3 beta (GSK-3β), and protease inhibitor cocktail (P8340) were obtained from Sigma-Aldrich (Merck KGaA, Darmstadt, Germany). Rabbit monoclonal anti-TNF-α (11948) was obtained from Cell Signaling Technology (Danvers, Massachusetts, USA). Anti-rabbit and anti-mouse horseradish peroxidase secondary antibodies (NA934V and NA931V, respectively) were obtained from GE Healthcare (UK). Clarity™ western enhanced chemiluminescence (ECL) substrate (Cat. #170–5061) and commercial kit DC™ Protein Assay (#5000112) were obtained from BIO-RAD Laboratories, Inc. (Hercules, CA, USA). The primary antibody polyclonal rabbit anti-glial fibrillary acidic protein (GFAP; Z0334) was obtained from Agilent Dako (CA, USA) and the primary polyclonal rabbit anti-pTau (pSER^396^; SAB4504557, phosphorylation site at serine 396) was obtained from Sigma-Aldrich (St. Louis, MO, USA). Histomount was obtained from National Diagnostics (Atlanta, GA, USA). All the other reagents used were obtained from Sigma-Aldrich (St. Louis, MO, USA) at the highest purity available.

### 2.2 Animals

Adult male CD-1 mice were 12 weeks old and weighed 38–48 g at the beginning of the experiment. They were purchased from Charles River Laboratories (L'Arbresle, France) and housed in IVC Sealsafe plus mouse Green Mouse 500 cages, in a temperature (22 ± 2 °C) and humidity-controlled environment, in a 12-h light-dark cycle. Standard rodent 4RF21 certificate diet (Mucedola, Settimo Milanese, Italy) and water were offered *ad libitum*. Each cage held a maximum of 3 animals to allow socialization and maintain animal welfare. Mice were weighed every day in the week before the first injection of DOX, to allow them to become accustomed to the handler, minimize stress, and improve animal welfare. Housing and experimental treatment of the animals were done following the guidelines defined by the European Council Directive (2010/63/EU) transposed into Portuguese law (Decreto-Lei no. 113/2013). The experiments were performed with the approval of the Portuguese National Authority for Animal Health (General Directory of Veterinary Medicine, process no. 0421/000/000/2016) and the local Committee Responsible for Animal Welfare (ICBAS-UP, ORBEA).

The schedule of DOX administration was designed to mimic human anticancer therapy, which consists of multiple administrations of the drug in cycles followed by drug-free periods [23]. Therefore, mice received intraperitoneal injections (i.p.) twice a week for three weeks. The animal received a total cumulative dose of 9 mg/kg (DOX 9) or 18 mg/kg (DOX 18) of DOX, which are equivalent to a human dose of 51.5 mg/m^2^ and 102.9 mg/m^2^, respectively [24,25], thus well below the maximum recommended dose. DOX was dissolved in a sterile saline solution (NaCl 0.9 %). The control group (CTRL) received injections of NaCl 0.9 % in the same volume and schedule as the drug-treated mice. Throughout the experimental period, the mice's well-being was monitored as described previously by the group [24,26]. After the last administration, the animals were kept drug-free for a week before euthanasia.

### 2.3 Samples collection

One week after the last administration, the mice were placed in a closed chamber in an atmosphere enriched with 5 % of the inhalant anaesthetic isoflurane until full sedation. When no reflex to stimuli was attained, exsanguination followed, with blood removed from the inferior vena cava. Afterwards, the mice were decapitated, and the brain was carefully removed and weighed. For some animals, the whole brain was homogenized in 5 % (w/v) HClO_4_ and centrifuged at 16,060 *g* for 10 min (4 °C). The supernatant was used for quantification of total glutathione (tGSH), reduced glutathione (GSH), oxidized glutathione (GSSG), and ATP, whereas the pellet was used for protein level determination. For Western blot analysis, brain hemispheres were homogenized in radio immune precipitation assay buffer (RIPA) and centrifuged at 16,060 *g* for 15 min (4 °C), as previously described [27]. Additionally, the brain hemispheres of the remaining animals were fixated in 4 % paraformaldehyde for histological processing.

### 2.4 Quantification of tGSH, GSH and GSSG

The tGSH and GSSG contents of the brain samples were quantified through the 5,5′-dithiobis(2-nitrobenzoic acid) (DTNB)-GSSG recycling assay, as previously described [28]. The levels of GSH were determined using the formula: GSH = tGSH – 2 x GSSG. The results of tGSH, GSH, and GSSG were normalized to the total protein content and expressed as a percentage of the control.

### 2.5 Quantification of brain ATP level

Whole brain levels of ATP were determined through the bioluminescent firefly luciferin-luciferase assay, as previously described [29]. The ATP levels were normalized to the total protein content and expressed as a percentage of the control.

### 2.6 Protein quantification

The protein contents of the collected brain samples were assayed with the commercial kit DC™ Protein Assay (Bio-Rad®, CA, USA), according to the manufacturer's recommendations and using bovine serum albumin (BSA) as a standard.

### 2.7 Western blot analysis

Following the paper by Dias-Carvalho, A. et al. [27], volumes of brain homogenate hemisphere samples equivalent to 50 μg or 70 μg of protein were reduced by Laemmli [30] buffer [0.5 M Tris-HCl pH 6.8, 4 % (w/v) SDS, 15 % (v/v) glycerol, 0.04 % (w/v) bromophenol blue and 20 % (v/v) β-mercaptoethanol] in a ½ (v/v) ratio, and then incubated at 100 °C for 5 min. A 12.5 % or 15 % SDS-polyacrylamide gel electrophoresis (SDS-PAGE) was used to perform electrophoresis at 180 V in running buffer [25 mM Tris, 192 mM glycine and 0.1 % (w/v) SDS] [30]. The blotting of the gels onto a nitrocellulose membrane (AmershamTM, Protan®, GE Healthcare) was performed for 2 h at 200 mA with transfer buffer (25 mM Tris, 192 mM glycine and 20 % methanol). As a protein loading control, a Ponceau S staining was performed. To block nonspecific binding, a solution of 5 % (w/v) nonfat dry milk in TBS-T (100 mM Tris, pH 8.0, 1.5 M NaCl and 0.5 % Tween 20) was used for 1 h at room temperature with agitation. The incubation for the ligation with the corresponding primary antibodies [anti-ATP synthase β (1:1000), anti-GSK-3β (1:1000), anti-SOD2/MnSOD (1:1000), and anti-HSP27 (1:500 and 1:1000)] was performed with constant agitation for 2 h at room temperature or overnight at 4 °C. Right after a washing step of 3 washes (10 min each) with TBS-T, the membranes were incubated with a secondary antibody, anti-rabbit (1:5000) or anti-mouse (1:1000) horseradish peroxidase, for 2 h at room temperature with agitation. A washing step followed, and then, according to the manufacturer's procedure, immunoreactive bands were detected with ECL reagents. The Gel Doc XR system (Bio-Rad®, CA, USA) was used to record and scan the images, which were analysed with Image Lab software (Bio-Rad®, CA, USA, version 6.0.1). The optical densities (OD) obtained were expressed in arbitrary units.

Mild stripping was performed when needed. This procedure included 2 x 10 min incubations with a stripping solution (0.04 M glycine, 0.7 mM SDS, 10 % Tween 20, pH 2.2), followed by two washing steps. The first included 2 × 10 min of washing with phosphate-buffered saline 1x (PBS) followed by a 5-min wash with TBS-T. After the stripping procedure, the blocking step was performed once again, and the membrane was ready for a new probe.

Finally, data analysis and normalization were done by the control bands. The average of control bands for each membrane was obtained and then divided by the smallest average of membrane control bands, resulting in a ratio for each membrane by which the remaining samples were divided. All results were divided by 1000 to work with smaller absolute values.

### 2.8 Brain tissue processing for histologic analysis

Left-brain hemispheres were labelled for blind analysis and processed according to a previously published method [27,31]. Succinctly, after a 24-h fixation in 4 % paraformaldehyde in 0.01 mM PBS (pH = 7.4) in low agitation at 4 °C, the hemispheres were switched to a 10 % sucrose solution diluted in PBS and kept overnight at 4 °C. The block of tissue obtained was then mounted on a Leica Vibratome VT1000S (Nuchloss, Germany) with the rostral surface up and consecutively sectioned in the coronal plane at 40 μm from the olfactory bulb to the caudal limit of the ventral hippocampus. The 40 μm sections were gathered in PBS and then kept at −20 °C in the Olmos cryoprotectant solution [30 % sucrose, 30 % ethylene glycol and 1 % polyvinylpyrrolidone-40 in PBS] until further processing.

### 2.9 Stereological analysis of the volume of the left HF

To identify the neuronal perikarya brain sections, they were stained with Giemsa solution following the protocol formerly published [27]. The volume of the left HF was assessed through the Cavalieri's Principle [32]. The Giemsa-stained sections were analysed using a modified Olympus BH-2 microscope interfaced with a colour video camera and equipped with a Heidenhain ND 281 microcator (Traunreut, Germany), a computerized part, and an object rotator (Olympus, Albertslund, Denmark). A computer equipped with a frame grabber (Screen Machine II, FAST Multimedia, Germany) was connected to the monitor. Based on Paxinos and Franklin's mouse brain atlas [33], the outline of the HF and its subregions (CA3, CA1, dentate gyrus (DG) and hilus) were marked using magnification of 100x. The volumes of the subregions CA1 and CA2 were determined together due to the difficulty in accurately determining the boundary between these subregions. Assessments were carried out using C.A.S.T. – grid system software (Olympus Albertslund, Denmark) and a mean of six systematically sampled sections containing dorsal HF area were used per animal. In each section, the cross-sectional area of each subregion was estimated by point counting [34] using an appropriate grid of test points. The area of the counting frame in the DG and Hilus was 15979.95 μm^2^, and in CA1 and CA3, it was 78913.35 μm^2^. The volume of each subregion was calculated from the total number of points that fell on each subregion and the thickness of the sections. The coefficient of error (CE) of the individual estimates was calculated according to Cruz-Orive [32] and the mean value was 0.10.

### 2.10 Immunohistochemistry

The determination of the total number of GFAP-immunoreactive (GFAP-ir) astrocytes was performed following the previously described method [27]. As a brief description, free-floating sections containing the HF region and sampled at regular intervals of 480 μm were washed with PBS ( 4 x 15 min) and treated with 10 % hydrogen peroxide (H_2_O_2_) in PBS for 10 min to inactivate endogenous peroxidase; and the sections were blocked with 5 % normal goat serum in PBS for 1 h at room temperature. Following this, the sections were incubated with the primary antibody polyclonal rabbit anti-GFAP at a 1:1000 dilution, for 72 h at 4 °C in PBS with 0.5 % Triton X-100. The secondary antibody, biotinylated goat IgG anti-rabbit antibody, was used at a 1:400 dilution for 1 h, followed by the incubation with the avidin-biotin peroxidase complex at a 1:800 dilution for 1 h. Afterwards, the sections were incubated for 1 min with 0.05 % 3,3′-diaminobenzidine (DAB), to which 0.01 % H_2_O_2_ was added and rinsed with PBS. The numerical density (Nv) of GFAP-ir astrocytes was estimated by applying the physical dissector method in an average of six systematically sampled sections per animal containing the dorsal hippocampal formation. Six to eight photographs per section were taken at a total magnification of 200x in a Zeiss scope A1 Axio microscope coupled with a coloured camera and a computer with the software Carl Zeiss AxioVision Rel. 4.8 (New York, USA). The images were uploaded to the software ImageJ 1.52a and the area of interest was delimited and the number of GFAP-ir astrocytes present in one focal plane and not in the other was counted. The thickness of the sections was also measured, using a microcator applied to the microscope, and the volume was determined using Cavalieri's Principle [32]. The total number of GFAP-ir astrocytes was calculated by multiplying Nv by the volume of the hippocampal formation.

Immunofluorescence detection of p53, pTau, Bax, Bcl-2, AIF and TNF-α was performed in sections containing the prefrontal cortex (PFC) or the HF, sampled at regular intervals of 480 μm (one out of 12). In detail, the previously sliced sections were washed with PBS (4 x 15 min ) to remove the Olmos solution and then incubated in 5 % normal horse serum in PBS with 0.25 % Triton X-100, for 1 h at room temperature to block nonspecific binding sites. Sections were incubated with the primary antibodies diluted in PBS with 0.25 % Triton X-100, for 72 h at 4 °C, in low agitation. The dilution ratio of primary antibodies was as follows: anti-p53 at a dilution of 1:250, anti-pTau at a dilution of 1:1500, anti-Bax at a dilution of 1:1000, anti-Bcl-2 at a dilution of 1:1000, anti-AIF at a dilution of 1:1000, and anti-TNF-α at a dilution of 1:500. The fluorescent secondary antibodies polyclonal anti-rabbit Alexa Fluor 488 (green; A32731) or polyclonal anti-rabbit Alexa Fluor 546 (red; A11035) were added to the samples at a dilution of 1:1000 in PBS with 0.25 % Triton X-100 and incubated for 1 h, at room temperature, protected from light. The slides were then washed in PBS (3 x 10 min ). The PBS-rinsed sections (3 x 10 min ) were cover slipped with FluorsafeTM mixed with DAPI at a 1:100 dilution. Photographs of p53, Bax and pTau immune stained sections were taken with identical exposure times, gain, and offsets for image acquisition in a Carl Zeiss Axio Imager 2.0 microscope coupled with a coloured camera and a computer with the software Carl Zeiss AxioVision Rel. 4.8 (New York, USA) for image capture. Images were obtained by manually scanning the PFC and the HF at a 20x magnification and at the following excitation/emission wavelengths: 358/461 (for DAPI), 500/525 (for Alexa Fluor 488) and 555/565 (for Alexa Fluor 546). For the measurement of intensity per area, the software ImageJ 1.52a was used.

### 2.11 Statistical analysis

Results are presented as mean ± standard deviation (SD). Statistical analysis was conducted using GraphPad Prism version 8.0.2 (GraphPad Software, CA, USA). Outliers were identified by the ROUT test (Q = 1 %) and removed before further statistical analyses. Statistical analysis between the three groups was done by a parametric analysis of variance (ANOVA), followed by the Tukey's *post hoc* test, once a significant *p* was achieved. Statistical significance was accepted at *p* < 0.05. When *p* > 0.05 but <0.1, a tendency was considered.

## 3 Results

### 3.1 Brain glutathione levels decreased in mice treated with the highest DOX dose, while MnSOD levels decreased with the lowest DOX dose

**Fig. 1:**
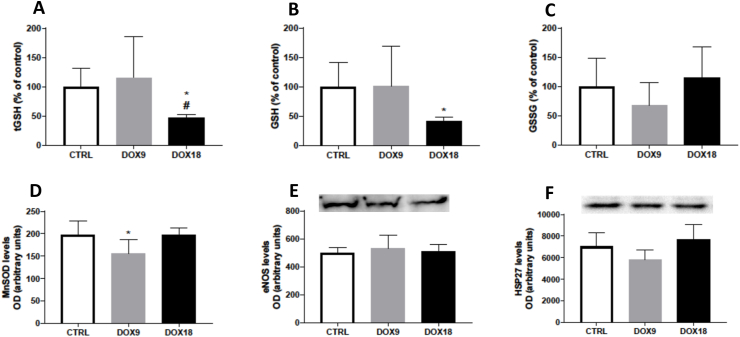
(A) Brain tGSH, (B) GSH, and (C) GSSG levels as % of control, are mean ± SD from 6 animals in each group, except in the control group that had 12 animals. (D) MnSOD (25 kDa), (E) eNOS (133 kDa), and (G) HSP27 (27 kDa) content were assessed by Western blotting and presented as optic density (OD). The results are expressed in arbitrary units, after DOX treatment from 5 to 10 biological duplicates from 4 to 6 independent animals. Data are represented as mean ± SD. Statistical comparisons were made using one-way ANOVA followed by Tukey's *post hoc* test (*p < 0.05 *vs.* control, CTRL; ^#^p < 0.05 *vs.* DOX9). Protein loading was confirmed by the Ponceau S staining (the original image and full blots are provided in the Supplementary data).

The highest dose of DOX significantly decreased the brain levels of tGSH and GSH, whereas the lower dose of 9 mg/kg had no significant impact on these measures (Fig. 1A and B). However, neither dose caused alterations in the GSSG levels (Fig. 1C). Regarding the Western blot determinations, MnSOD levels were reduced with the lowest cumulative DOX given (Fig. 1D) and no significant changes were observed in the eNOS and HSP27 levels with either dose (Fig. 1E and F).

### 3.2 The lowest dose of DOX tended to decrease brain ATP synthase β content

**Fig. 2:**
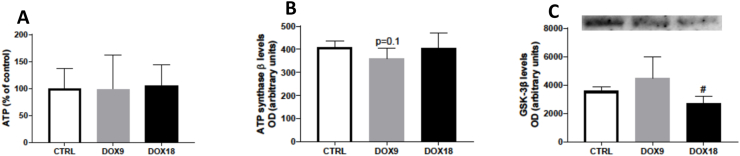
Brain (A) ATP levels as % of mean control, are mean ± SD from 6 animals in each group, except in the control group that had 12 animals. Levels of (B) ATP synthase β (52 kDa) and (C) GSK-3 β (50 kDa), as assessed by Western blotting and presented as optic density (OD). These latter results are expressed in arbitrary units, after DOX treatment from 5 to 10 biological duplicates from 4 to 6 independent animals. Data are represented as mean ± SD. Statistical comparisons were made using one-way ANOVA followed by Tukey's *post hoc* test (^#^p < 0.05 *vs.* DOX9). Protein loading was confirmed by the Ponceau S staining (the original image and full blots are provided in the Supplementary data).

None of the administered doses of DOX caused alterations in the overall brain ATP levels (Fig. 2A). Nonetheless, the cumulative dose of 9 mg/kg caused a tendency (*p* = 0.1) to decrease the brain levels of ATP synthase β when compared to control, with no changes seen for the highest dose (Fig. 2B). The levels of GSK-3 β had no changes after DOX when compared to control animals (Fig. 2C).

### 3.3 The highest dose of DOX increased the hippocampal formation volume in mice

**Fig. 3:**
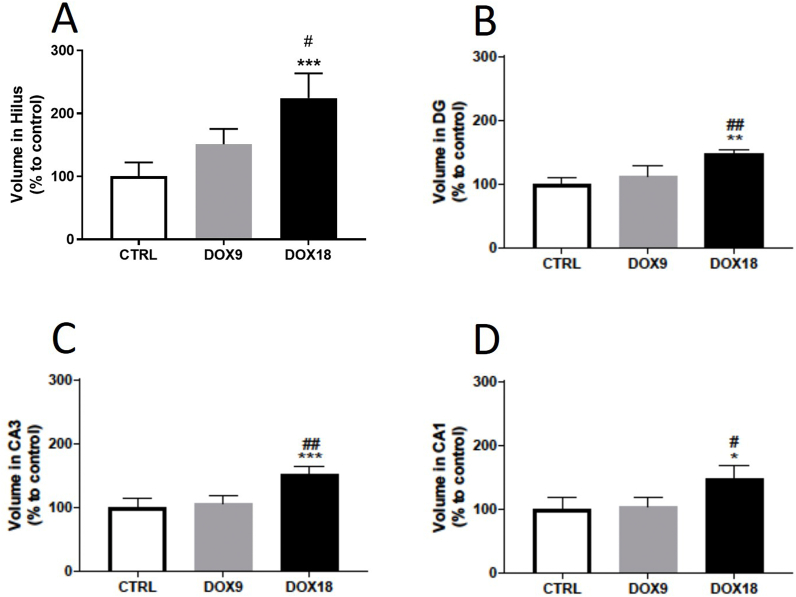
Volume in brain hippocampal regions (A) Hilus, (B) DG, (C) CA3 and (D) CA1 in mice brain exposed to a total cumulative dose of 9 mg/kg DOX (DOX9) or a total cumulative dose of 18 mg/kg DOX (DOX18). Data, as % of mean control, are expressed by mean ± SD from 4 animals in each group. Statistical comparisons were made using one-way ANOVA followed by Tukey's post hoc test (*p < 0.05, **p < 0.01, ***p < 0.001 *vs.* control, CTRL; ^#^p < 0.05, ^##^p < 0.01 *vs.* DOX9).

Considering the hippocampal formation volume, the highest total cumulative dose of 18 mg/kg DOX caused a significant increase in hippocampal volume in all regions evaluated (Fig. 3A–D), when compared to the control group and the lowest DOX dose group. That increase was more noticeable in the Hilus region (Fig. 3A). The lowest DOX dose did not induce volume alterations in any of the evaluated hippocampal brain regions (Fig. 3A–D).

### 3.4 DOX increased GFAP-ir astrocytes in the CA3 region, but decreased them in the DG hippocampal region

**Fig. 4:**
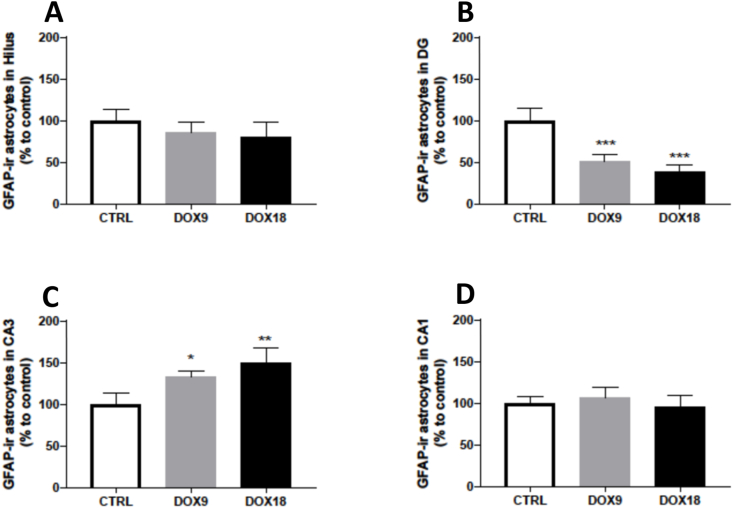
GFAP-immunoreactive astrocytes (GFAP-ir astrocytes) in the hippocampal formation regions (A) Hilus, (B) DG, (C) CA3 and (D) CA1 in mice brain exposed to a total cumulative dose of 9 mg/kg DOX (DOX9) or a total cumulative dose of 18 mg/kg DOX (DOX18). Data, as % of mean control, are expressed as mean ± SD from 4 animals in each group. Statistical comparisons were made using one-way ANOVA followed by Tukey's *post hoc* test (*p < 0.05, **p < 0.01, ***p < 0.001 *vs.* control, CTRL).

None of the DOX cumulative doses given altered the estimated number of total GFAP-ir astrocytes in the Hilus (Fig. 4A) or in the CA1 brain regions of mice (Fig. 4D). However, both the lower (9 mg/kg) and higher (18 mg/kg) DOX doses decreased GFAP-ir astrocytes in the DG (Fig. 4B). Importantly, there was a substantial increase in the CA3 region of GFAP-ir astrocytes evoked by both DOX doses (Fig. 4C).

### 3.5 DOX caused dissimilar changes in Bax expression, depending on dosing and brain area

**Fig. 5:**
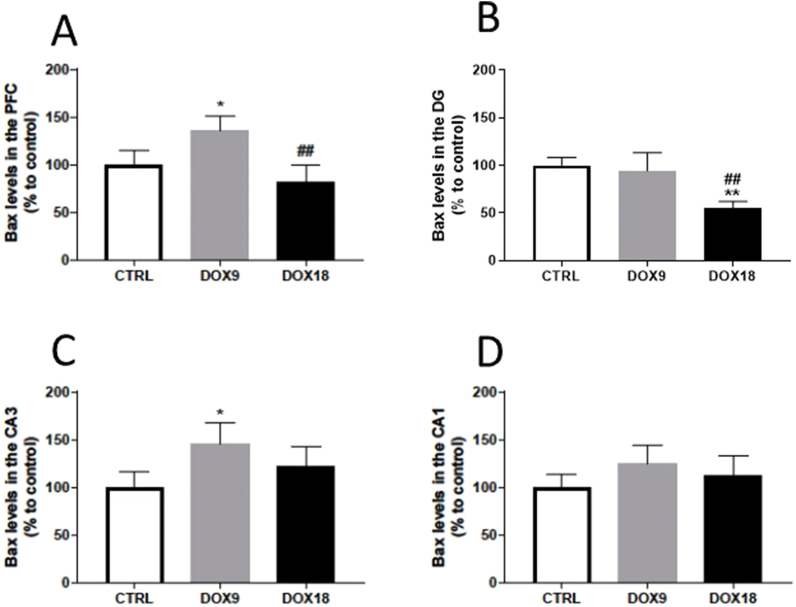
Bax levels in the (A) PFC, and hippocampal formation regions (B) DG, (C) CA3 and (D) CA1 in mice brain exposed to a total cumulative dose of 9 mg/kg DOX or a total cumulative dose of 18 mg/kg DOX. Results, as % of mean control, are expressed as mean ± SD from 4 animals in each group. Statistical comparisons were made using one-way ANOVA followed by Tukey's *post hoc* test (*p < 0.05 and **p < 0.01 *vs.* control, CRTL, ^##^p < 0.01 *vs.* DOX9).

Regarding the markers of cell death signalling, the administration of a total cumulative dose of 9 mg/kg DOX led to a significant increase in Bax expression in the PFC when compared to the saline control, whereas the higher dose of DOX (18 mg/kg) caused a significant decrease in Bax expression in comparison to the 9 mg/kg DOX-treated animals (Fig. 5A). Regarding hippocampal formation, the total cumulative dose of 18 mg/kg DOX diminished the expression of this pro-apoptotic protein when compared to both the saline control and the lower dose of DOX (Fig. 5B). Nevertheless, in the CA3 region, the administration of 9 mg/kg DOX led to an increase in Bax expression in comparison to the control group (Fig. 5C). The CA1 region did not present any meaningful alterations in Bax expression in any of the DOX doses administered (Fig. 5D).

### 3.6 The highest dose of Dox decreased Bcl-2 levels in the HF

**Fig. 6:**
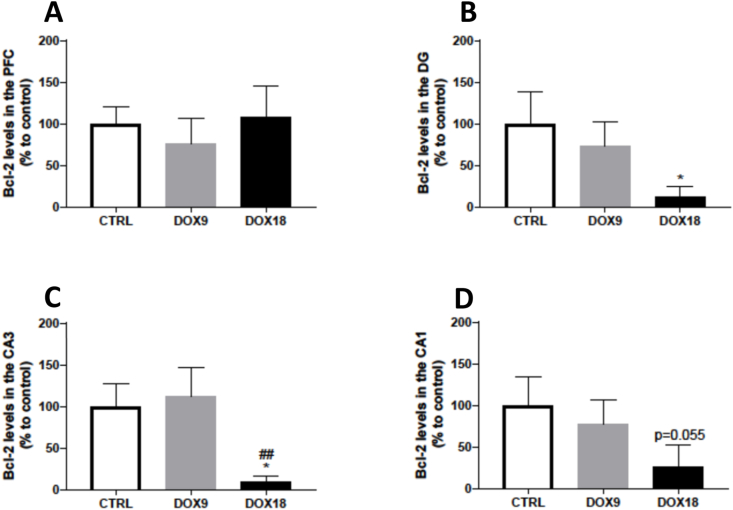
Bcl-2 levels in the (A) PFC, and hippocampal formation regions (B) DG, (C) CA3 and (D) CA1 in mice brain exposed to a total cumulative dose of 9 mg/kg DOX or a total cumulative dose of 18 mg/kg DOX. Results, as % of mean control, are expressed as mean ± SD from 3 animals in each group. Statistical comparisons were made using one-way ANOVA followed by Tukey's *post hoc* test (*p < 0.05 *vs.* control, CRTL, ^##^p < 0.01 *vs.* DOX9).

The expression of the anti-apoptotic protein Bcl-2 was not altered in the PFC by any of the administered doses (Fig. 6A). However, Bcl-2 levels decreased significantly in all HF regions of the mice treated with a total cumulative dose of 18 mg/kg, whereas the total cumulative dose of 9 mg/kg did not affect the Bcl-2 levels in these regions (Fig. 6B–D).

### 3.7 The lowest dose of DOX increased p53 expression in the CA3 region

**Fig. 7:**
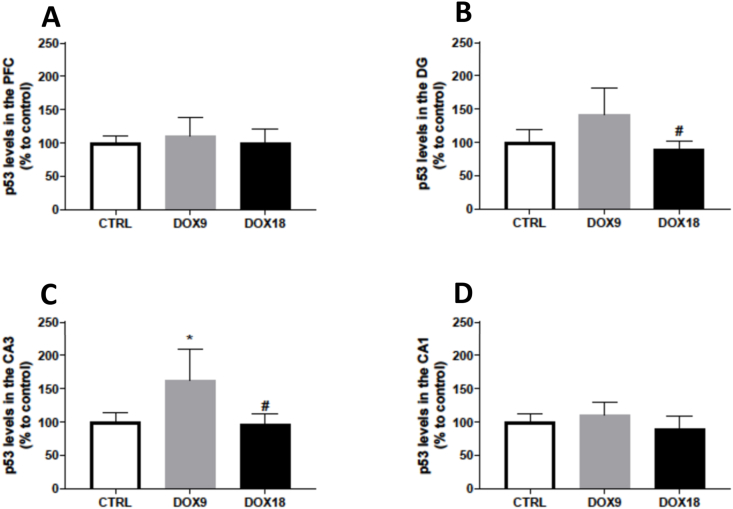
p53 levels in the (A) PFC, and hippocampal formation regions (B) DG, (C) CA3 and (D) CA1 in mice brain exposed to a total cumulative dose of 9 mg/kg DOX or to a total cumulative dose of 18 mg/kg DOX. Data, expressed as % of mean control, are represented as mean ± SD from 4 animals in each group. Statistical comparisons were made using one-way ANOVA followed by Tukey's *post hoc* test (*p < 0.05 *vs.* control, CTRL, ^#^p < 0.05 *vs.* DOX9.

Neither the lower (9 mg/kg) nor the higher (18 mg/kg) total cumulative dose of DOX affected the expression of p53 in the PFC area (Fig. 7A). The treatment with 18 mg/kg DOX induced a decreased expression of p53 when compared to the lower DOX dose in the DG and CA3 of the hippocampal formation (Fig. 7B and C), but no significant changes were seen in comparison to control animals. In contrast, the total cumulative dose of 9 mg/kg increased p53 expression in the CA3 region when compared to the control (Fig. 7C). Meanwhile, the CA1 region was unaffected by any of the DOX doses administered (Fig. 7D), as far as p53 was concerned.

### 3.8 AIF levels increased significantly in the DG in mice administered with 18 mg/kg DOX

**Fig. 8:**
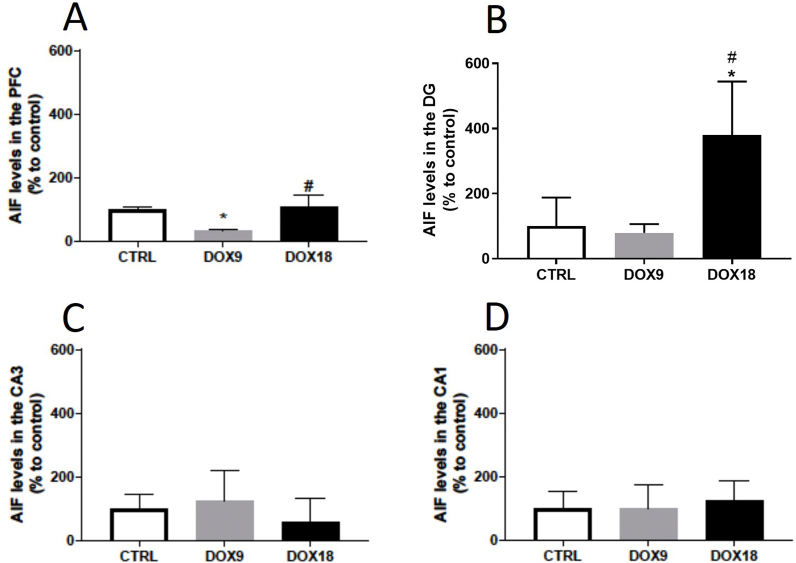
AIF levels in the (A) PFC, and hippocampal formation regions (B) DG, (C) CA3 and (D) CA1 in mice brain exposed to a total cumulative dose of 9 mg/kg DOX or to a total cumulative dose of 18 mg/kg DOX. Data, as % of mean control, are represented as mean ± SD from 3 animals in each group. Statistical comparisons were made using one-way ANOVA followed by Tukey's *post hoc* test (*p < 0.05 *vs.* control, CTRL, ^#^p < 0.05 *vs.* DOX9).

AIF content decreased in the PFC in mice that received the lowest dose (9 mg/kg) of DOX, whereas at the highest dose (18 mg/kg) there were no differences when compared to controls (Fig. 8A). In the DG region, AIF increased considerably in the 18 mg/kg treated mice in comparison with both the saline control and the lowest DOX dose (Fig. 8B). In the CA3 and CA1 regions, no significant alterations were observed (Fig. 8C and D).

### 3.9 Mice given the dose of 18 mg/kg DOX had less pTau in the DG and CA3 regions

**Fig. 9:**
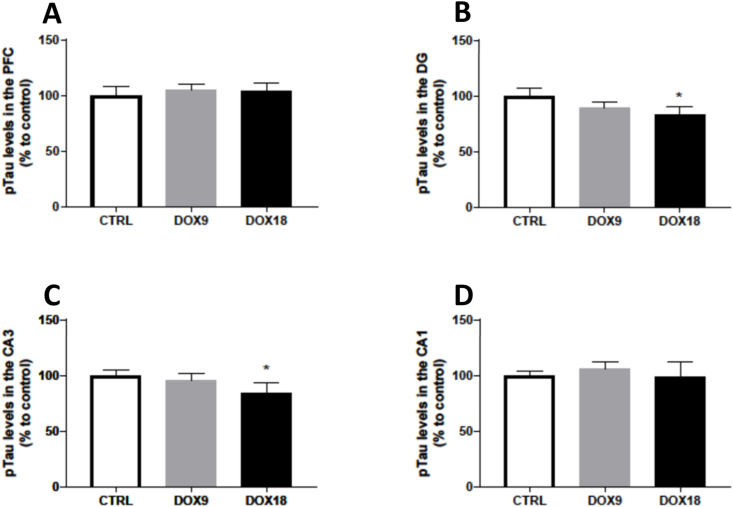
pTau levels in the (A) PFC, and hippocampal formation regions (B) DG, (C) CA3 and (D) CA1 in mice brain exposed to a total cumulative dose of 9 mg/kg DOX or a total cumulative dose of 18 mg/kg DOX. Data, as % of mean control, are presented as mean ± SD from 4 animals in each group. Statistical comparisons were made using one-way ANOVA followed by Tukey's *post hoc* test (*p < 0.05 *vs.* control, CTRL).

None of the administered doses of DOX altered the levels of phosphorylated Tau protein in the PFC (Fig. 9A). In the hippocampal formation, the administration of a total cumulative dose of 18 mg/kg caused a decrease in pTau levels in the DG and CA3 regions in comparison to the saline controls (Fig. 9B and C), whereas DOX did not evoke changes in the CA1 region (Fig. 9D).

### 3.10 TNF-α levels increased in the CA1 region in the animals treated with the highest dose

**Fig. 10:**
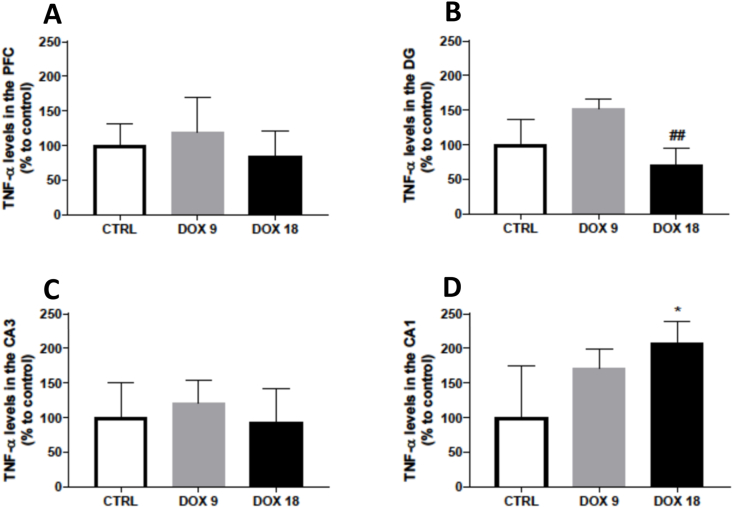
TNF-α expression in the (A) PFC, and hippocampal formation regions (B) DG, (C) CA3 and (D) CA1 in mice brain exposed to a total cumulative dose of 9 mg/kg DOX or a total cumulative dose of 18 mg/kg DOX. Data, as % of mean control, are presented as mean ± SD from 4 animals in each group. Statistical comparisons were made using one-way ANOVA followed by Tukey's *post hoc* test (*p < 0.05 *vs.* control, CTRL, ^##^p < 0.05 *vs.* DOX9).

None of the administered doses caused significant changes in TNF-α levels in either the PFC or CA3 region (Fig. 10A and C). The highest dose of DOX caused an elevation of TNF-α levels in the CA1 region (Fig. 10D). In the DG region, the mice treated with 18 mg/kg DOX had a decrease in TNF-α levels in comparison to the mice treated with 9 mg/kg DOX (Fig. 10B).

## 4 Discussion

Chemotherapy-inflicted adverse effects lead to high morbidity, and as the number of cancer survivors increases, concern about the long-term consequences of chemotherapy is becoming more relevant [4]. While the BBB has several mechanisms that protect the brain from DOX access, it seems that this chemotherapeutic agent can damage the brain through still not fully disclosed mechanisms. The major findings of this work were the following: the highest tested dose of DOX disrupts the antioxidant defences and increases the volume of all brain areas evaluated; GFAP-ir astrocytes decreased in the DG and increased in the CA3 region in a dose-dependent manner; regarding apoptotic markers, DOX 18 caused changes in Bax, Bcl-2 and AIF levels in several brain regions; furthermore, in some brain areas, the highest DOX cumulative dose decreased pTau and increased TNF-α levels.

The brain has a high oxygen consumption, a high lipid-rich environment, and a poor antioxidant content, making it highly susceptible to oxidative stress [35]. The antioxidant GSH is vital for redox balance. It acts as an antioxidant by directly binding with electrophiles or by eliminating peroxides with the concomitant formation of the dimeric oxidized form GSSG [36]. In this study, 18 mg/kg of DOX caused brain oxidative stress, as seen by a decrease in the brain levels of tGSH and GSH. These changes were not accompanied by changes in GSSG levels. Moreover, the dose of 9 mg/kg DOX did not cause any significant alteration in the glutathione parameters, suggesting a dose-dependent action of DOX. Studies in male mice (2–3 months old) treated with a single i.p. dose of 20 mg/kg DOX and sacrificed 72 h later, reported an increase in whole brain GSH levels [17]. A single dose of 25 mg/kg DOX also significantly raised brain levels of GSH and increased GSSG levels [15], showing that brain glutathione equilibrium is changed after DOX. Despite not altering the glutathione parameters, only the 9 mg/kg cumulative dose of DOX caused significant reductions in the whole brain MnSOD content, which suggests that different doses affect the antioxidant system in different manners. MnSOD is responsible for modulating ROS concentration in the mitochondria [37,38], and a lower DOX exposure seems to compromise its synthesis or turnover, despite no changes in glutathione levels observed. Wahdan et al. [39] also reported a decrease in MnSOD levels in the hippocampal tissue after administration of DOX (8 mg/kg cumulative dose, i.p.), in accordance with our data. In our research, the expression of eNOS did not suffer significant changes with DOX treatment. eNOS is an enzyme responsible for the generation of the majority of ^●^NO in endothelial cells. This enzyme has several functions, namely the control of blood vessel tone, angiogenesis, and it is responsible for the majority of the ^●^NO levels in the hippocampus and the cortex [[40], [41], [42]]. Despite a study finding higher levels of ^●^NO in brain homogenates [43], it does not seem to be due to higher eNOS content, but rather a possible increase in other isoforms of ^●^NO synthase or a byproduct of redox reactions. HSP27 levels were also determined since this protein is able to respond to cellular stress conditions, such as oxidative stress, working as an antioxidant in several key events [44]. DOX did not alter the expression of HSP27 in total brain homogenates. As far as we know, the literature does not include reports about the expression of HSP27 itself in the brain after the administration of DOX. However, Simončíková et al., observed no changes in the levels of HSP27 in the heart of male Wistar rats after the administration of DOX (15 mg/kg, i.p.) [45]. Our data in the brain corroborate their results, and therefore, as far as we can infer from the present studies, these cumulative doses of DOX do not promote changes in HSP27 levels.

Besides whole brain analysis, an in-depth immunohistochemistry analysis was performed to evaluate several biomarkers in the PFC and HF, since these brain areas are involved in important processes of memory and cognition, which have been reported in clinical studies to be often affected by chemobrain [[46], [47], [48]]. Firstly, the volume of the HF was assessed through Cavalieri's Principle in Giemsa-stained sections. Starting from the edge of the “C-curve” of this structure is the DG, where neuronal stem cells give rise to neurons in the adult brain [49]. It presents three distinct layers, an outer molecular layer, a middle granule cell layer, and an inner polymorphic layer also known as the hilus, followed by the cornu ammonis subfields: CA3, CA2, CA1 [50]. In this evaluation, the total cumulative dose of 18 mg/kg DOX increased hippocampal formation volume in all of the regions evaluated with the most pronounced increase being seen in the hilus, which is the inner polymorphic layer of the DG [51]. Based on the data from the literature available, there are no reports of chemotherapy-treated patients or animals under study displaying an increase in hippocampal volume. Nevertheless, hippocampal volumetric enlargement can happen due to cytotoxic oedema, which consists of swelling of brain cells that precedes the onset of cell death [52]. Moreover, oxidative stress has been reported to be involved in increased vascular permeability and oedema [53]. As previously mentioned, DOX is a well-characterized inducer of oxidative stress and, in this study, the total cumulative dose of 18 mg/kg DOX-induced oxidative stress in adult mice's brain, which can relate to these results.

Astrocytes are the largest and most abundant glial cells, outnumbering neurons by five times [54]. They support neuronal function in a variety of ways, such as homeostatic regulation, and increasing synaptic plasticity and are involved in the neuroinflammatory process [53]. Upon brain damage, mature astrocytes become more reactive by releasing mainly neurotrophic factors, inflammatory factors and cytotoxins. Those changes are accompanied by morphological changes such as increased and longer processes and thicker and larger cellular bodies. Furthermore, increased astrocyte proliferation and increased GFAP expression correlate with reactive astrogliosis [55,56]. Given that neuroinflammatory processes have been associated with chemotherapy-induced cognitive dysfunction, the evaluation of the total number of GFAP-ir astrocytes was made in the HF. The PFC was not analysed since the technique used requires precise delimitation of the structure and the limits in the PFC are not as defined as in the HF. The lowest and highest total cumulative doses of DOX caused the same alteration patterns: the total number of GFAP-ir astrocytes decreased in the DG whereas in the CA3 region an increased number was seen. The pronounced decrease in the DG is most likely due to both increased astrocyte death and decreased proliferation caused by the DOX insult. While there is no data reporting GFAP-ir decreases in the DG due to DOX administration, a reduced number of GFAP-ir in the DG was found in individuals with major depressive disorder [57]. The effects of DOX administration are further seen in the CA3 region with the increase in reactive astrocytes. Similar results were obtained in the hippocampus of rats administered i.p. with a total cumulative dose of 8 mg/kg DOX over 4 weeks [58]. All animals in that study had considerable impaired cognitive function in domains dependent on proper hippocampal function, similar to what is observed in cancer patients treated with regimens that include DOX [59]. This corroborates that our experimental model has clinical relevance to highlight the brain's adverse outcome pathways of chemotherapy.

Considering that DOX induces cell apoptosis in other non-target tissues [60], several apoptotic markers were evaluated in both the PFC and HF. Bax is an apoptosis-promoting factor of the Bcl-2 family, usually located in the cytosol. Upon an apoptotic stimulus, it promotes cell death by suffering conformational changes, resulting in consequent translocation to the mitochondria and insertion in the outer mitochondrial membrane. This translocation results in a subsequent pore formation and cytochrome *c* leakage to the cytosol culminating in irreversible apoptosis [61]. DOX effects on Bax content were dose-dependent, meaning that the total cumulative dose of 9 mg/kg DOX increased Bax expression in the PFC and CA3 regions, while the total cumulative dose of 18 mg/kg DOX decreased Bax expression in the PFC and DG regions. Bax increased expression was observed in mice administered with a total cumulative dose of 8 mg/kg DOX for 4 weeks [62], suggesting apoptosis activation. Herein, however, probably the highest dose of DOX caused a similar increase in Bax expression earlier in the experiment, or most likely, elicited other mechanisms of cellular death. Dual-way cell death depending on the DOX concentration was reported in an *in vitro* study where cortical neurons exposed to lower concentrations of DOX (up to 0.5 μM) elicited apoptotic death whereas for higher concentrations, apoptosis was inhibited and necrosis became dominant [63]. The anti-apoptotic protein Bcl-2 was also determined. Bcl-2 action is repressed by Bax through the formation of Bcl-2/Bax heterodimers and this interaction commands cell apoptosis [64]. In this study, the DOX18 group had significantly lower levels of Bcl-2 in all the HF subregions evaluated, further suggesting that, at the studied time-point and in higher doses, other mechanisms of cell death might be involved or apoptosis might occur earlier. Additionally, the tumour suppressor p53 was also quantified to better understand the mechanisms at play. Among the many cellular processes controlled by this factor, p53 mediates cellular apoptosis by activating the proapoptotic proteins of the Bcl-2 family, such as Bax, to permeabilize the mitochondrial membrane [64]. The 9 mg/kg DOX group had increased p53 levels in the CA3 region, while the highest dose did not cause any significant changes. Considering the influence of p53 on Bax activation, it is important to note that Bax expression also increased in 9 mg/kg DOX-treated mice. The lowest DOX dose induced an increase in both p53 and Bax content (although no changes in Bcl-2 were seen) in the CA3 region, indicating that apoptotic death might be occurring in this region. On the contrary, in the DG and CA3 of the DOX18 group, Bax decreased and p53 only decreased in comparison to the DOX9 group, which indicates that apoptotic death might have happened at another time-point before the mice's sacrifice or necrosis became the major cell death pathway. Supporting this notion is the study of Tangpong et al., where a single i.p. dose of 20 mg/kg DOX increased p53 expression 3 h after DOX administration, accompanied by Bax increase, indicating apoptotic cell death. Nonetheless, 24 h after DOX, the levels returned to normal, due to a compensating increase in anti-apoptotic factors such as Bcl-xL, which promotes cell survival [15]. However, with the total cumulative dose of 18 mg/kg DOX used in the present study, it seems that there was no attempt to promote cell survival as seen by the marked decrease in Bcl-2 content.

AIF, a caspase-independent element of the apoptotic cascade located in the mitochondrial intermembrane space was also evaluated. The obtained results showed an increase on AIF content in the DG of the DOX18 group. In contrast, a decrease was observed in the PFC of the DOX9 group. Despite no reports, as far as we know, on the effects of DOX in AIF brain levels, a study has shown that this protein is involved in neuronal apoptosis and mediates neuronal death [65]. Therefore, the data suggest that, at higher cumulative doses of DOX, the AIF apoptotic route might be involved in neuronal death.

In the PFC and the HF, phosphorylated Tau content was measured. This microtubule-associated protein is mostly expressed in the axons of neurons and its main functions are the stabilization of microtubule bundles and control of microtubule assembly, dynamic behaviour, and spatial organization. pTau has been described as one of the key markers for Alzheimer's disease and ageing brain, being responsible for paired helical filament formation once hyperphosphorylated [66]. The highest dose of DOX induced a decrease in the pTau expression in the DG and CA3 regions. One possible explanation is that the Tau-expressing cells such as neurons are less present, or other internal mechanisms of cellular regulation are occurring, rendering a decrease in pTau expression.

Finally, TNF-α has been widely studied as it could be the bridge between the peripheral toxicity of DOX and neuronal toxicity [15]. In our study, only the highest cumulative dose (DOX18) caused a significant increase of TNF-α in the CA1 region. Studies reported that DOX increased TNF-α content in whole-brain homogenates [15] and in hippocampal homogenates [39], however, to the best of our knowledge, TNF-α levels in the different HF subregions have not been reported. Due to the heterogeneous nature of this brain structure, the DOX-toxic insult may affect brain areas in different manners, which need to be further disclosed.

## 5 Conclusion

The main goal of this study was to assess the effects of two different clinically relevant doses of DOX in the brains of male CD-1 mice. Despite many studies on the neurotoxicity of DOX, many fail to mimic real clinical scenarios using single drug administrations, instead of cycles of chemotherapy administration. At the time-point studied, both tested doses caused neurotoxicity; however, in slightly different manners. Conversely, both doses induced extensive astrogliosis. Our work also demonstrated that the different brain areas react differently to the DOX-toxic insult, and the use of brain/hippocampal homogenates might dilute some of the results. Particularly in the hippocampal formation, the DG and CA3 subregions were the most affected by DOX. The DG has the particularity of being a site of adult neurogenesis and could be more susceptible to the neurotoxicity of DOX. Furthermore, this subregion projects to the CA3 subfield through the mossy fibre pathway, connecting both structures and possibly extending the damage from one to another. Overall, as expected, the higher DOX dose was more neurotoxic since it caused oxidative stress and an increase in the hippocampal volume that was not present in the lower dose. On the other hand, one cannot overrule the putative long-term effects of even the smallest DOX doses. The observed changes may be possible causes for the cognitive impairments observed in treated patients, but further studies are needed to evaluate if these changes are irreversible.

## Funding

This work is financed by national funds from Fundação para a Ciência e a Tecnologia (10.13039/501100001871FCT), I.P., in the scope of the project UIDP/04378/2020 and UIDB/04378/2020 of the Research Unit on Applied Molecular Biosciences (UCIBIO) and the project LA/P/0140/2020 of the Associate Laboratory Institute for Health and Bioeconomy—i4HB and through the project EXPL/MEDFAR/0203/2021. A. Dias-Carvalho acknowledges FCT and 10.13039/501100015621UCIBIO for her PhD grant (UI/10.13039/100017412BD/151318/2021). V.M.C acknowledges 10.13039/501100001871FCT for her grant (SFRH/BPD/110001/2015) that was funded by national funds through 10.13039/501100001871FCT under the Norma Transitória – DL57/2016/CP1334/CT0006. A.R.-M. acknowledges 10.13039/501100001871FCT for her grant SFRH/10.13039/100017412BD/129359/2017.

## Data availability statement

The data presented in this study are available on request from the corresponding authors.

## Institutional review board statement

The study was conducted according to the guidelines of the Declaration of Helsinki and approved by the Ethics Committee of the local animal welfare body (ICBAS-UP ORBEA) and the Portuguese national authority for animal health (DGAV, process no. 0421/000/000/2016).

## CRediT authorship contribution statement

**Ana Dias-Carvalho:** Writing – original draft, Investigation, Formal analysis. **Mariana Ferreira:** Writing – review & editing, Investigation. **Ana Reis-Mendes:** Writing – review & editing, Investigation. **Rita Ferreira:** Writing – review & editing, Supervision, Conceptualization. **Maria de Lourdes Bastos:** Writing – review & editing, Supervision. **Eduarda Fernandes:** Writing – review & editing, Supervision, Investigation. **Susana Isabel Sá:** Writing – review & editing, Validation, Supervision, Investigation, Conceptualization. **João Paulo Capela:** Writing – review & editing, Supervision, Investigation. **Félix Carvalho:** Writing – review & editing, Supervision, Investigation. **Vera Marisa Costa:** Writing – review & editing, Supervision, Investigation, Conceptualization.

## Declaration of competing interest

The authors declare the following financial interests/personal relationships which may be considered as potential competing interests: Vera Marisa Costa is part of Heliyon's Editorial Board.
