# Supplementary material for: Doxorubicin-induced neurotoxicity differently affects the hippocampal formation subregions in adult mice
Source: Heliyon. 2024 May 24;10(11):e31608. doi: 10.1016/j.heliyon.2024.e31608 (PMC11168325; doi:10.1016/j.heliyon.2024.e31608)
Supplement: Multimedia component 1 [file mmc1.docx]

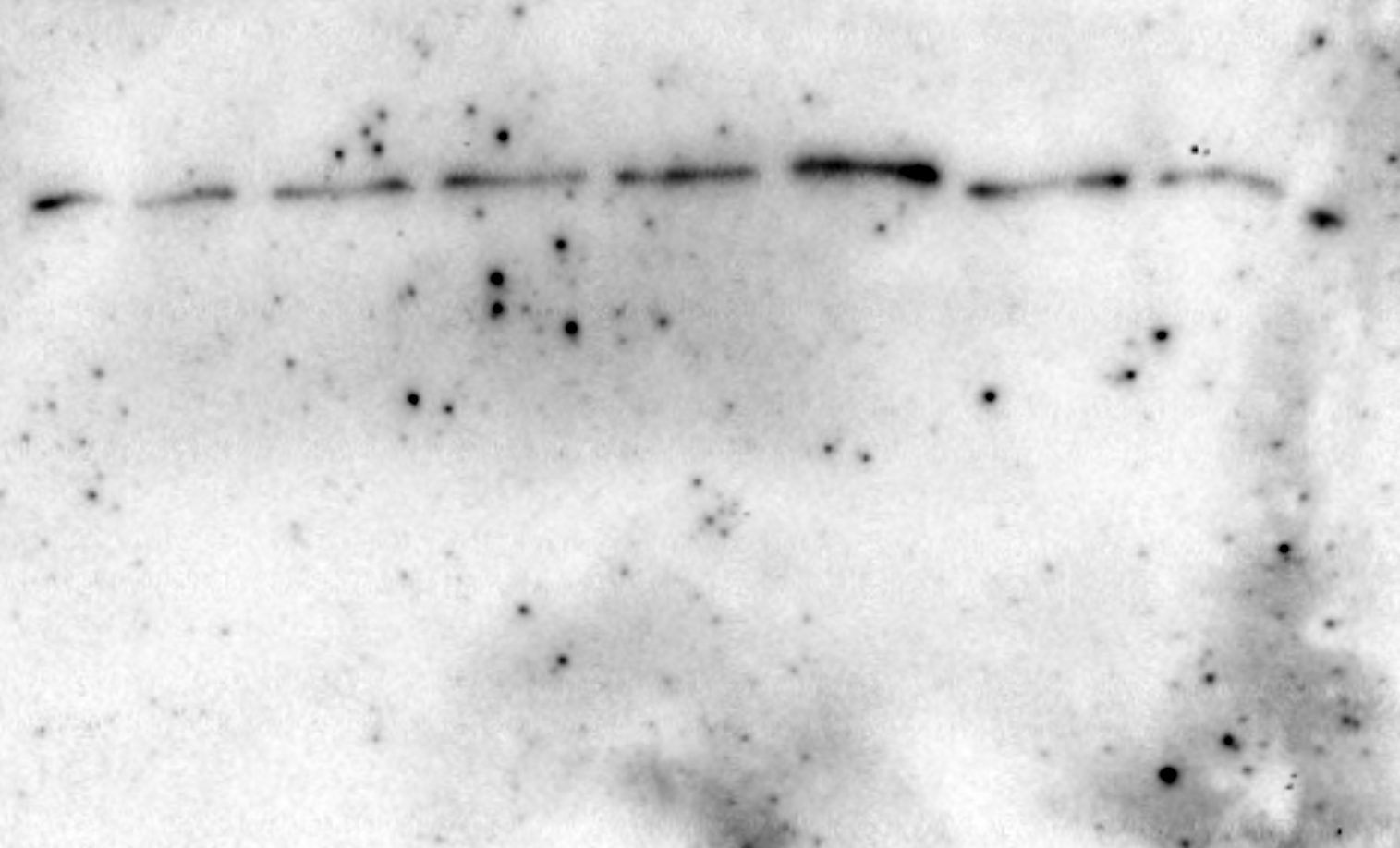


C

D9

C

D9

D18

**C**


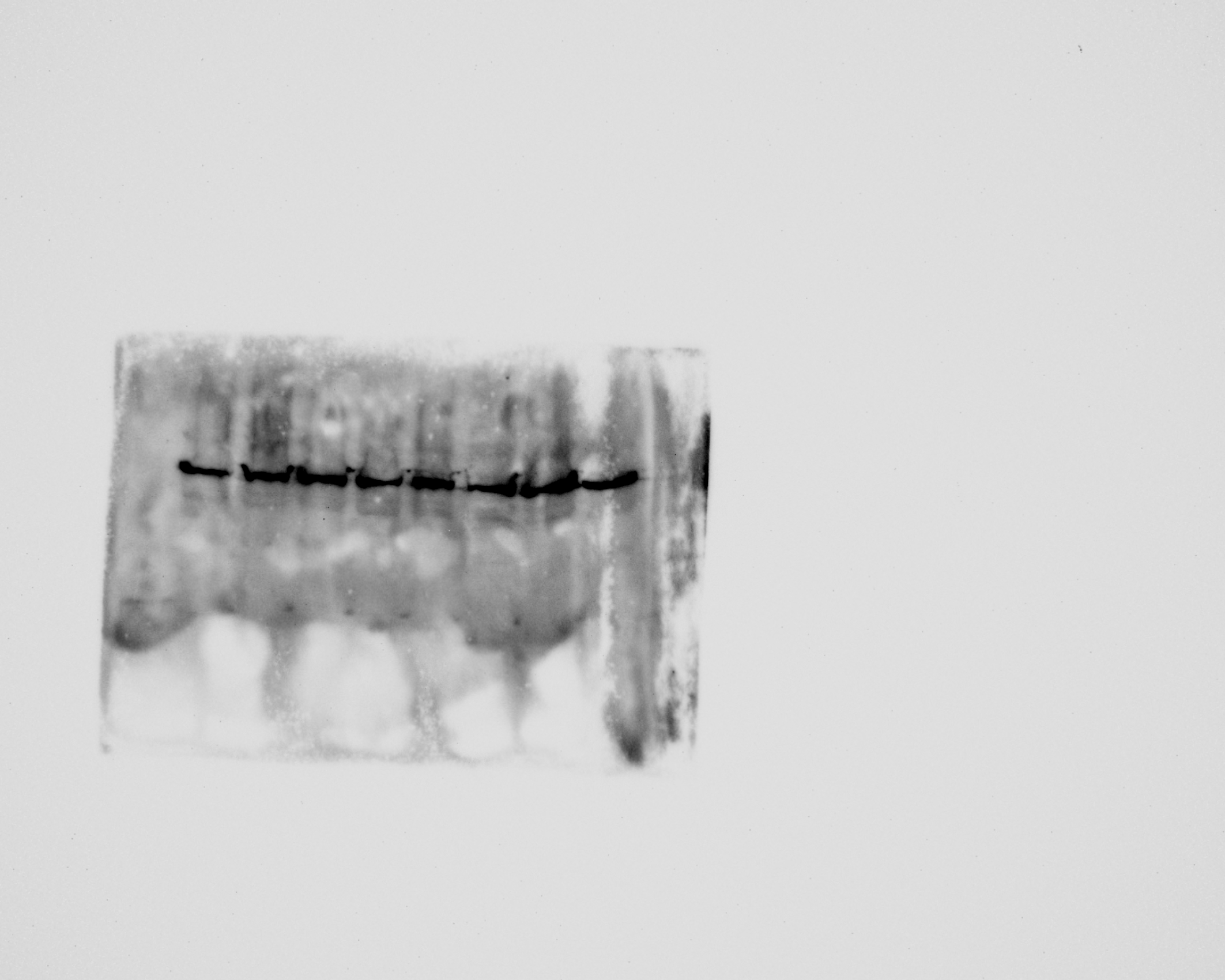


C

D9

D18

C

D9

D18

**B**


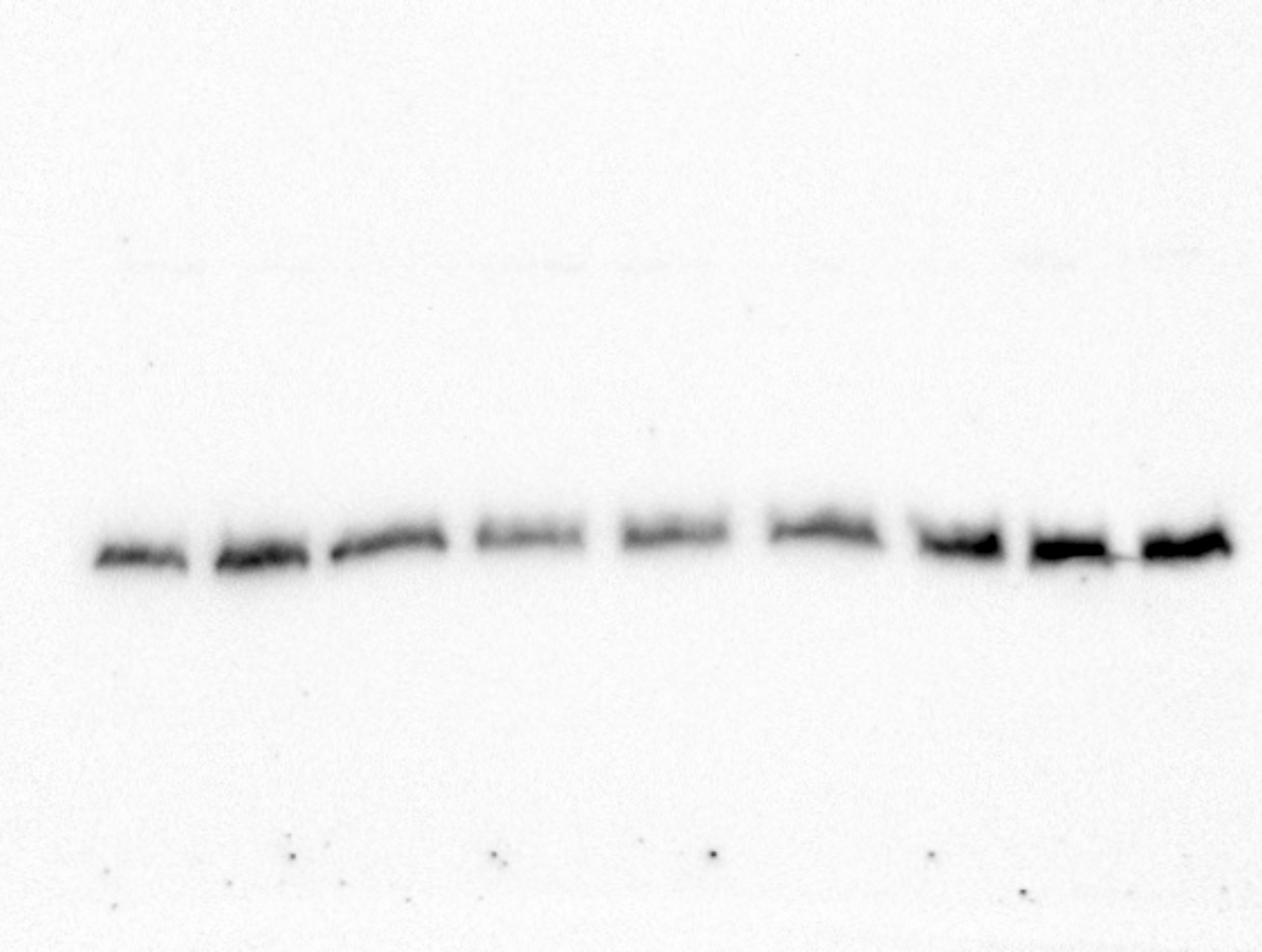


C

D9

**A**

C

D9

C

D18

Full length bands of blots of the expression of (A) MnSOD; (B) eNOS; C Hsp27; (D) ATP synthase β; (E) GSK-3β. (C – Control; D9 - Doxorubicin 9mg/kg; D18 – Doxorubicin 18 mg/kg.)

C


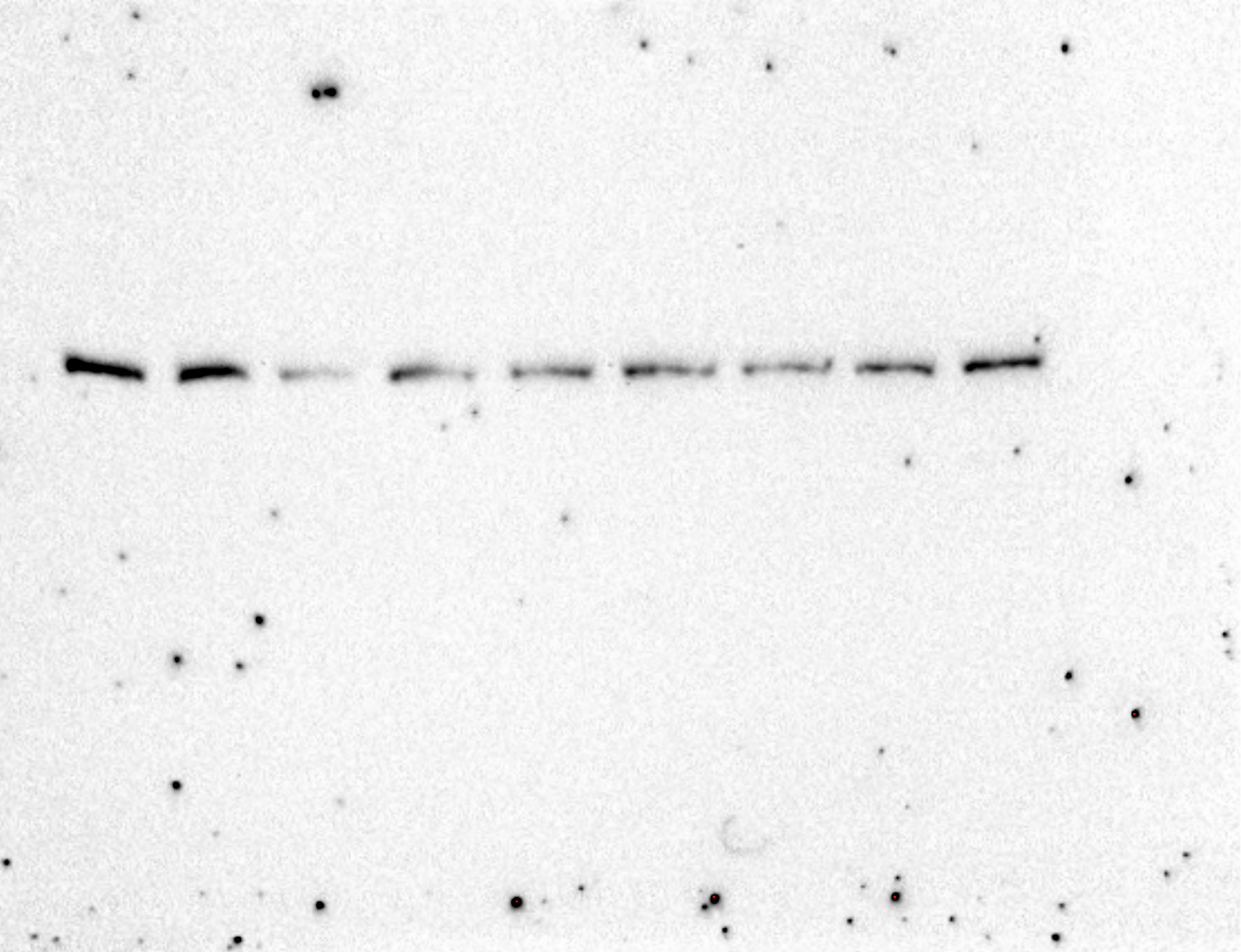


C

D9

C

D18

C

**D**

C

D9


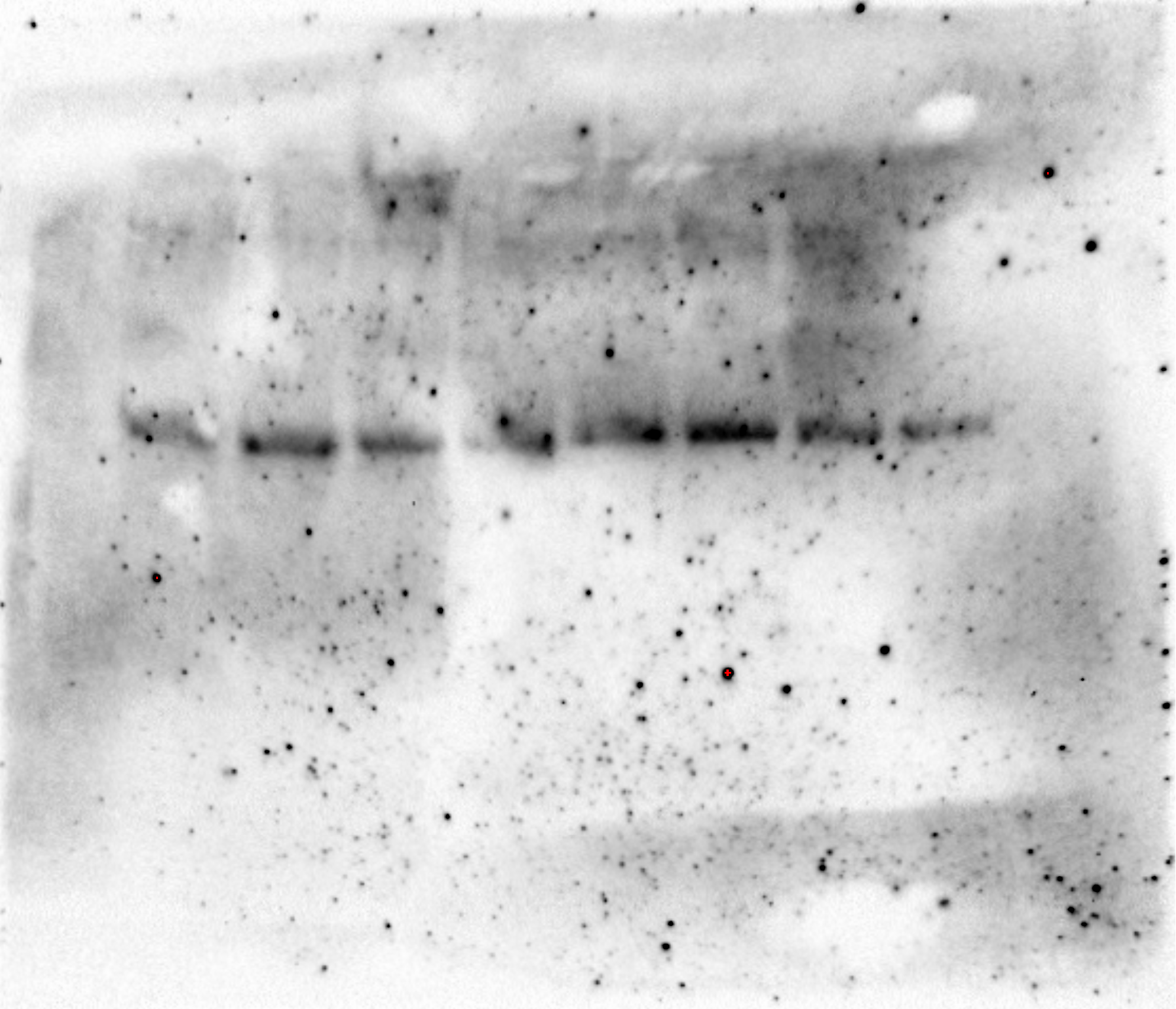


C

D9

D18

C

D9

D18

**E**


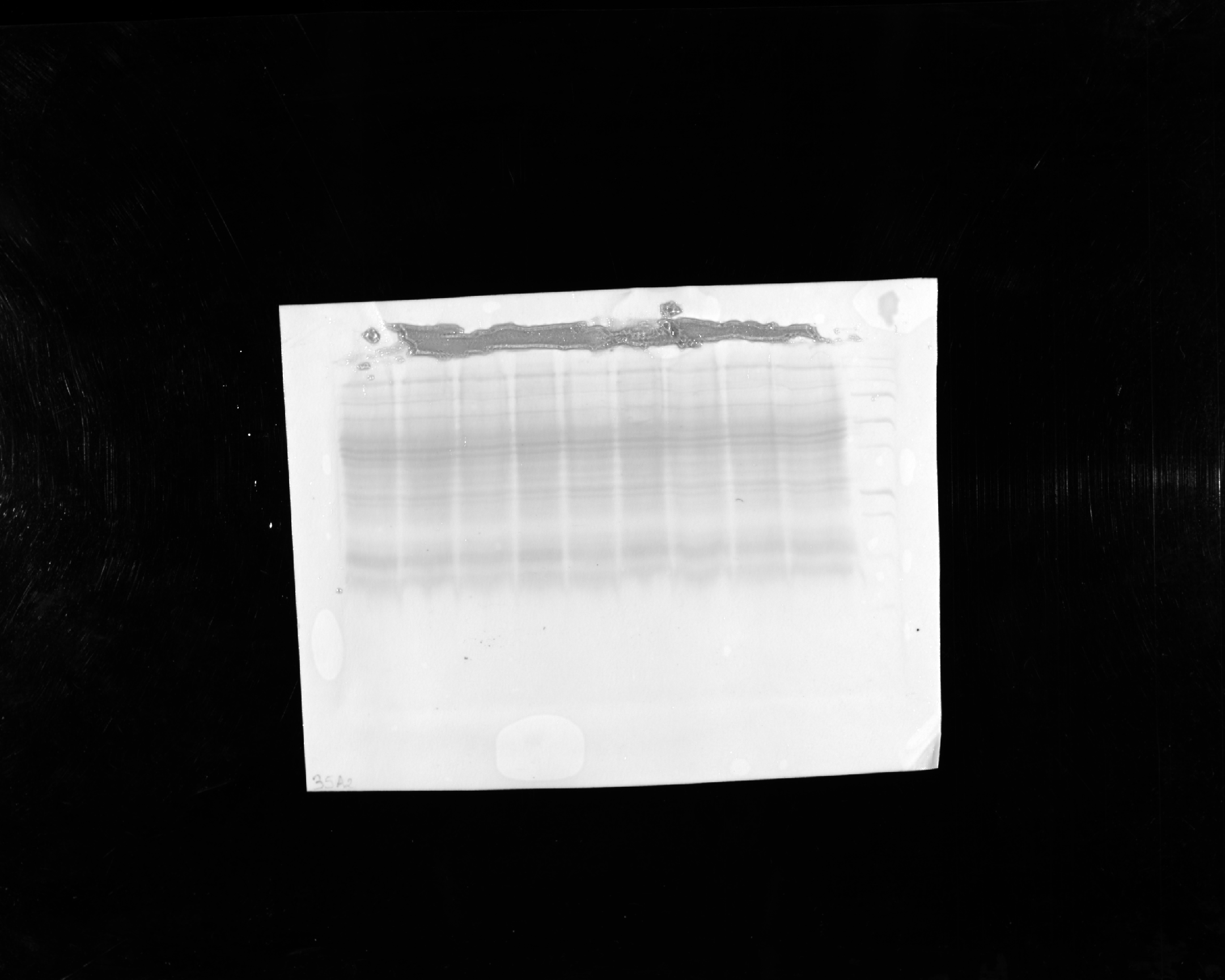

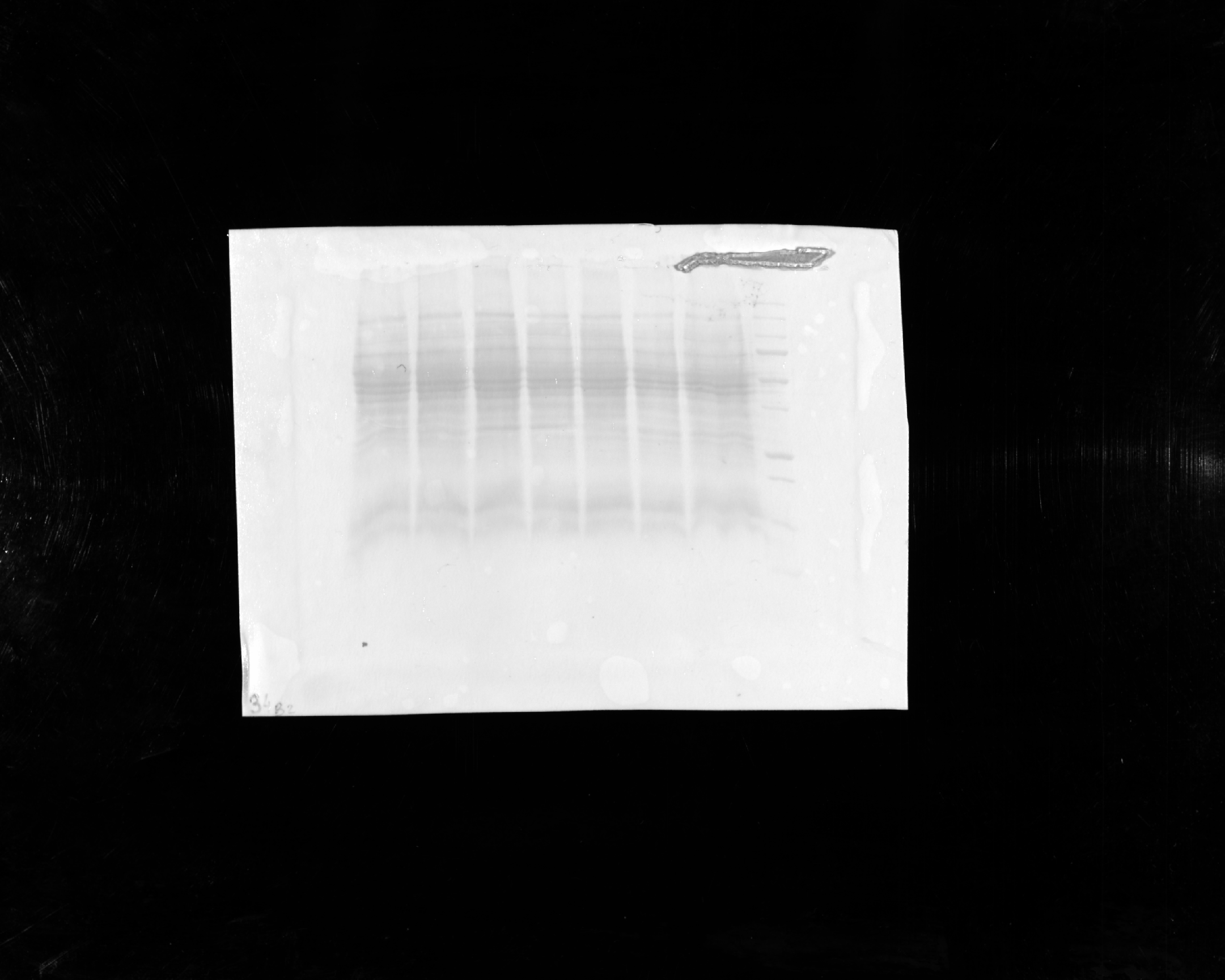

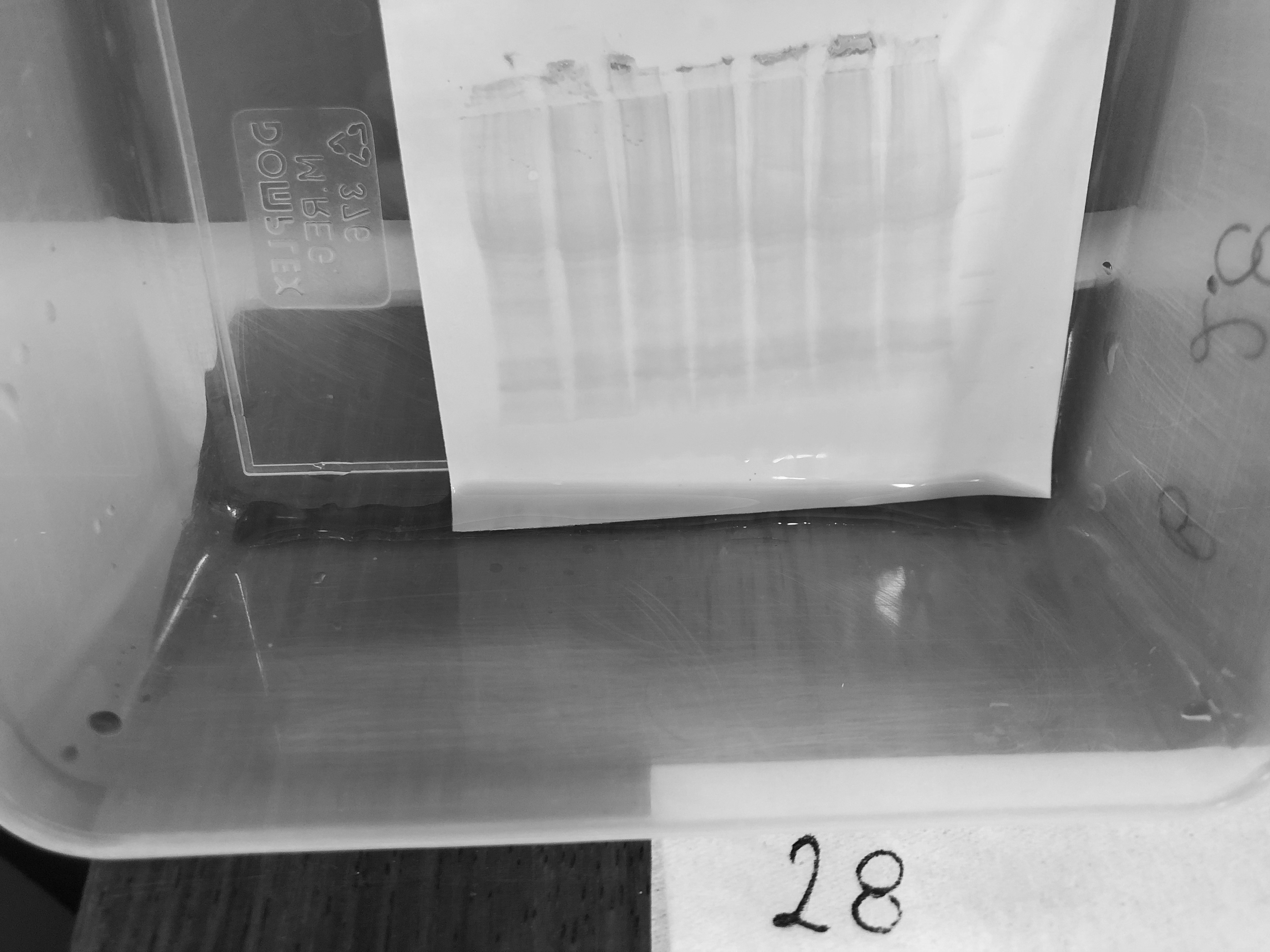

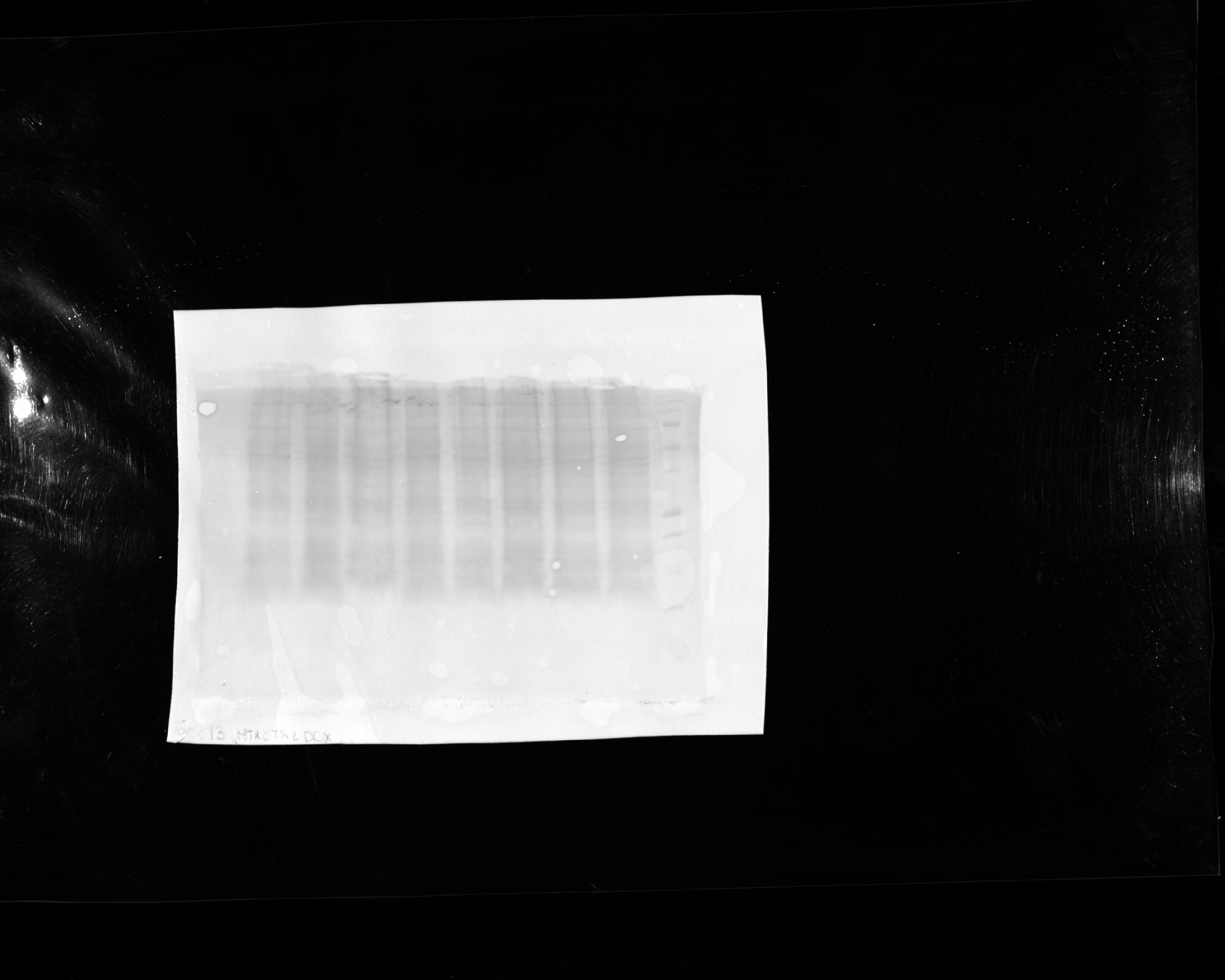

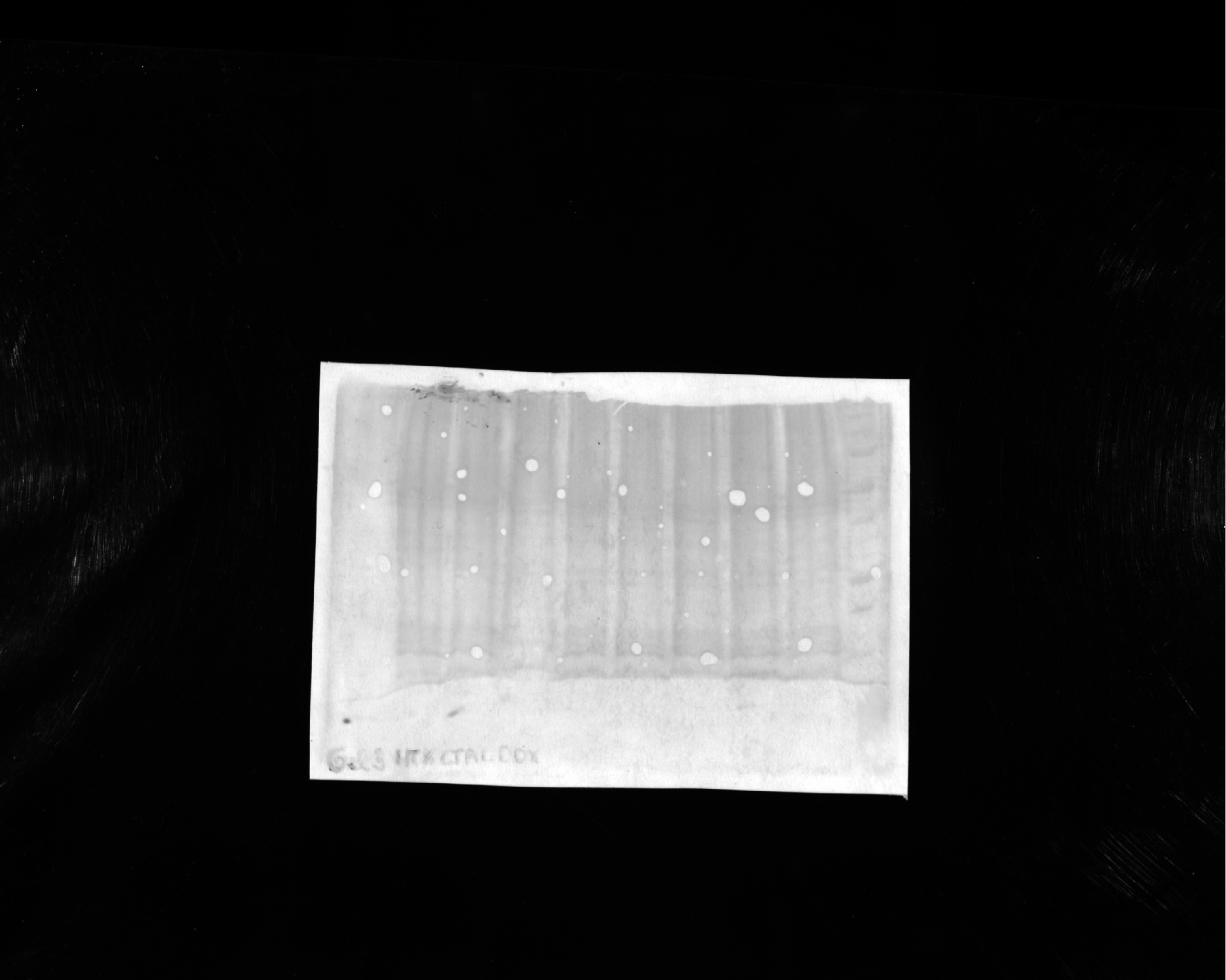

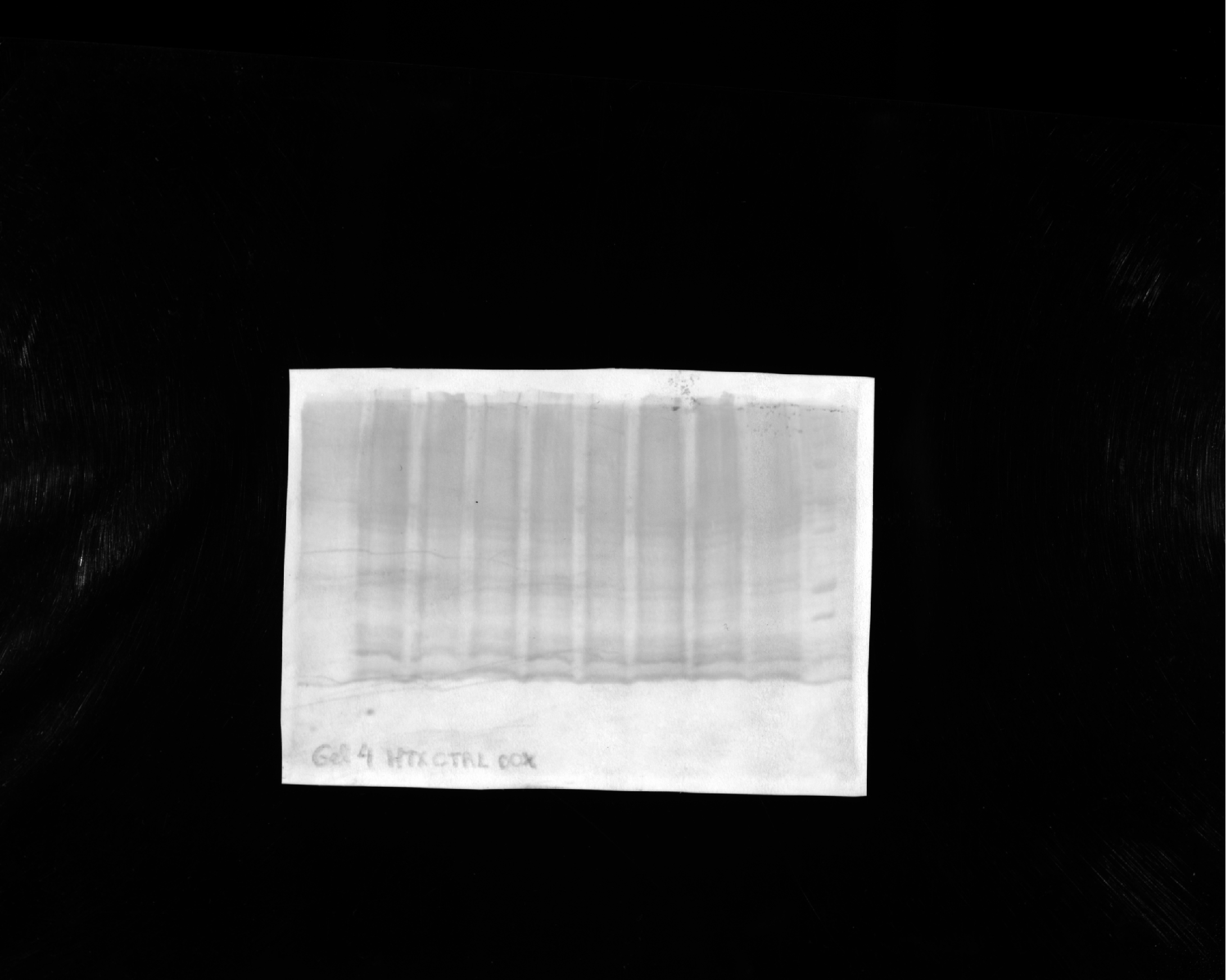

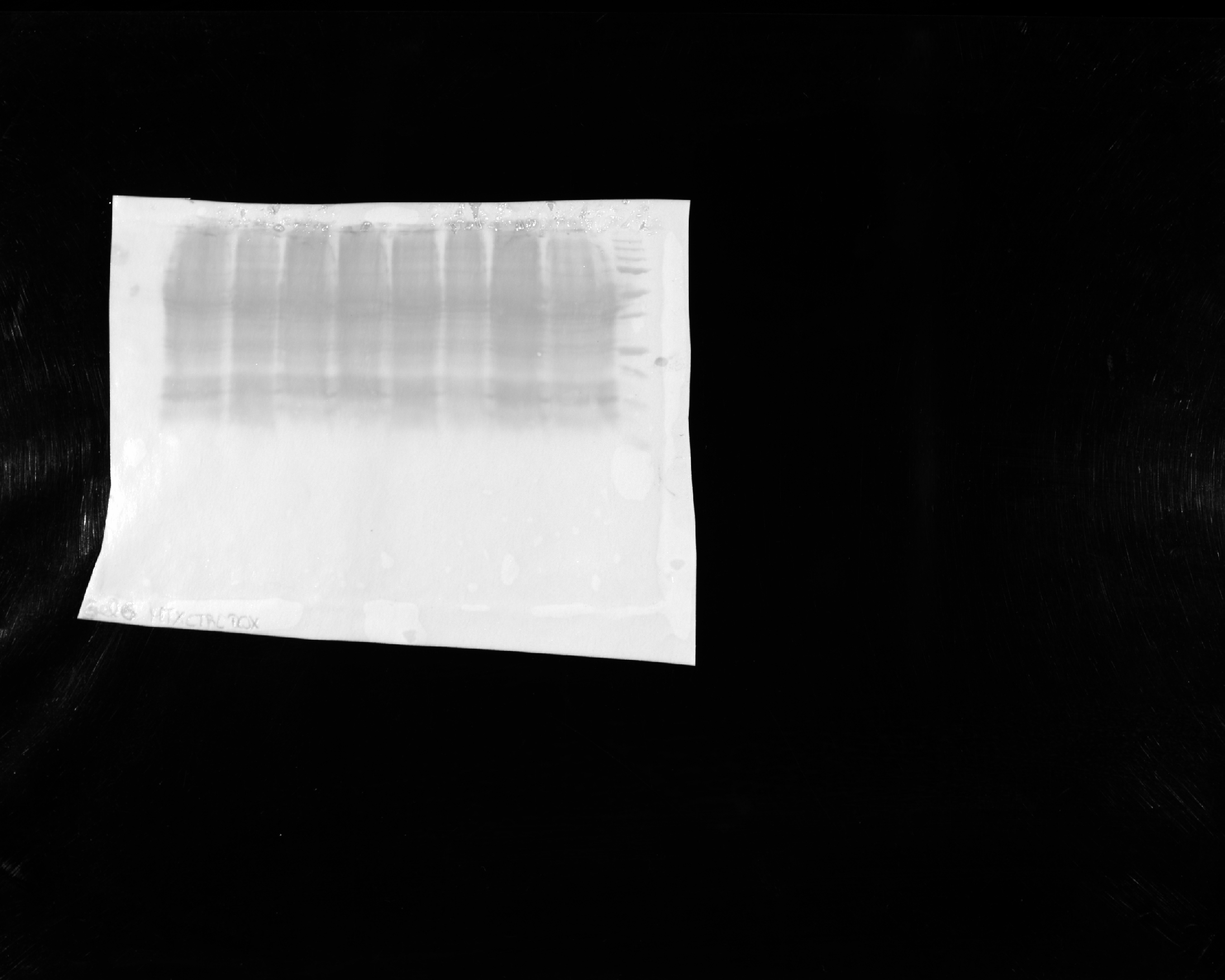


Loading control of Ponceau S staining taken of gels used for (A) MnSOD (25 kDa), (B) eNOS (133 kDa), (C) eNOS (133 kDa), (D) ATP synthase B (52 kDa), (E) GSK-3B (50 kDa), (F) HSP27 (27 kDa), and (G) GSK-3B (50 kDa).

**E**

**A**

**B**

**C**

**D**

**F**

**G**
